# Supplementary material for: Synthesis of Second-Generation Analogs of Temporin-SHa Peptide Having Broad-Spectrum Antibacterial and Anticancer Effects
Source: Antibiotics (Basel). 2024 Aug 11;13(8):758. doi: 10.3390/antibiotics13080758 (PMC11350846; doi:10.3390/antibiotics13080758)
Supplement: Supplementary file 1 [file antibiotics-13-00758-s001.zip › antibiotics-3094880-supplementary.pdf]

Synthesis of Second-Generation Analogs of Temporin-SHa Peptide Having Broad-Spectrum Antibacterial and Anticancer Effects

Arif Iftikhar Khan [1], Shahzad Nazir [1], Muhammad Nadeem ul Haque [1], Rukesh Maharjan [1], Farooq-Ahmad Khan [1], Hamza Olleik [2], Elise Courvoisier-Dezord [2], Marc Maresca [2,*] and Farzana Shaheen [1,*]

[1] Third World Center for Science and Technology, H. E. J. Research Institute of Chemistry, International Center for Chemical and Biological Sciences, University of Karachi, Karachi 75270, Pakistan; khanformanite@gmail.com (A.I.K.); nazirshahzad39@gmail.com (S.N.); nadeem.and.chem@gmail.com (M.N.u.H.); rukeshmaharjan2013@gmail.com (R.M.); farooq.khan@iccs.edu (F.-A.K.)

[2] Aix Marseille Univ, CNRS, Centrale Med, ISM2, 13013 Marseille, France; hamza.olleik@live.com (H.O.); elise.courvoisier-dezord@univ-amu.fr (E.C.-D.)

* Correspondence: m.maresca@univ-amu.fr (M.M.); afnan.iccs@gmail.com (F.S.); Tel.: +33-0413945609 (M.M.); +92-3313859073 (F.S.)

Table of Contents

#	Content	Page No.
S1	UPLC profile of temporin-SHa peptide (**1**), [G10a]-SHa peptide (**2**) and its second-generation analogs (**3–6**) in ACN : H_2O : TFA (60 : 40 : 0.08) at 215 nm	3–4
S2	Synthetic scheme of temporin-SHa peptide (**1**)	5
S3	HR-MS (MALDI) spectrum of temporin-SHa (**1**)	6
S4	^{1}H-NMR of temporin-SHa (**1**)	7
S5	NMR data of temporin-SHa (**1**) in d_6-DMSO	8–9
S6	Synthesis of [G10a]-SHa (**2**)	10
S7	LR-ESI-MS of [G10a]-SHa (**2**)	11
S8	^{1}H-NMR of [G10a]-SHa (**2**)	12
S9	NMR Data of [G10a]-SHa in d_6-DMSO (**2**)	13–15
S10	Synthesis of [G10f]-SHa (**3**)	16
S11	LR-ESI-MS of [G10f]-SHa (**3**)	17
S12	^{1}H-NMR spectrum of [G10f]-SHa (**3**)	18
S13	NMR data of [G10f]-SHa (**3**) in d_6-DMSO	19–21
S14	Synthesis of [G10K]-SHa (**4**)	22
S15	LR-ESI-MS of [G10K]-SHa (**4**)	23
S16	^{1}H-NMR spectrum of [G10K]-SHa (**4**)	24
S17	NMR Data of [G10K] SHa (**4**) in d_6-DMSO	25–27
S18	Synthesis of [G10n]-SHa (**5**)	28
S19	LR-ESI-MS of [G10n]-SHa (**5**)	29
S20	^{1}H-NMR spectrum of [G10n]-SHa (**5**)	30
S21	NMR data of [G10n]-SHa (**5**) in d_6-DMSO	31–33
S22	Synthesis of [G10y]-SHa (**6**)	34
S23	LR-ESI-MS of [G10y]-SHa (**6**)	35
S24	^{1}H-NMR spectrum of [G10y]-SHa (**6**)	36
S25	NMR data of [G10y]-SHa in d_6-DMSO (**6**)	37–38
S26	UV-Vis. spectra of peptide-analogs (**1–6**)	39
S27	FT-IR spectra of peptide-analogs (**1–6**)	40

UPLC Profile of temporin-SHa and its analogs in ACN:H_2O:TFA (60:40:0.08) at 215 nm

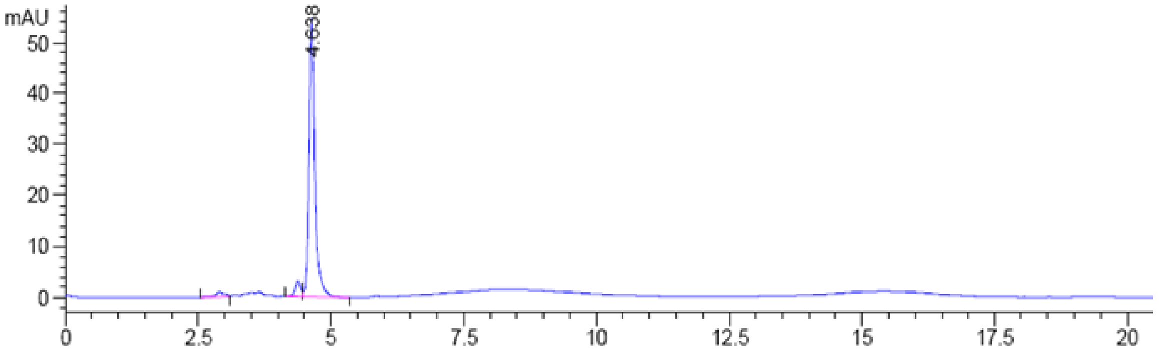

UPLC profile of temporin-SHa (**1**) at C-4 reverse-phase column, ACN:H_2O:TFA (60%:40%:0.082%)

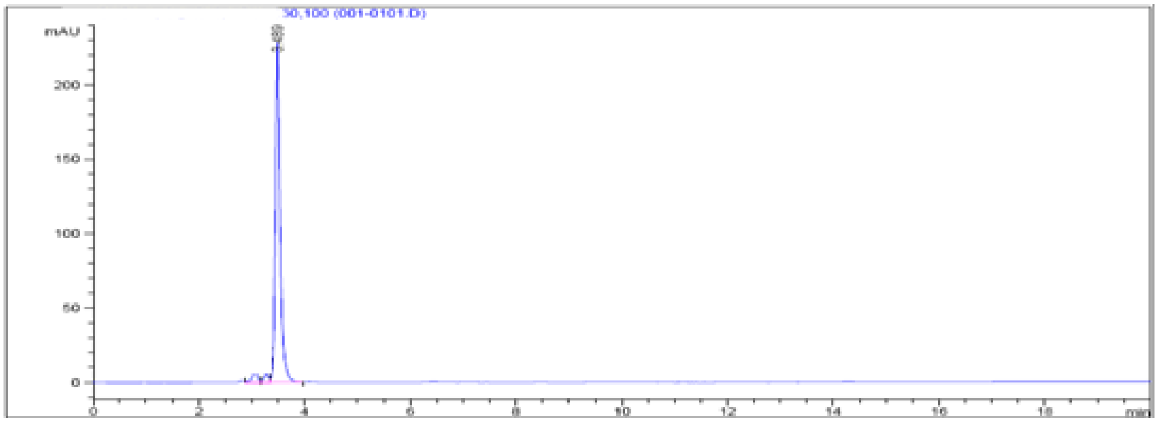

UPLC profile of [G10a]-SHa (**2**) at C-4 reverse-phase column, ACN:H_2O:TFA (60%:40%:0.082%)

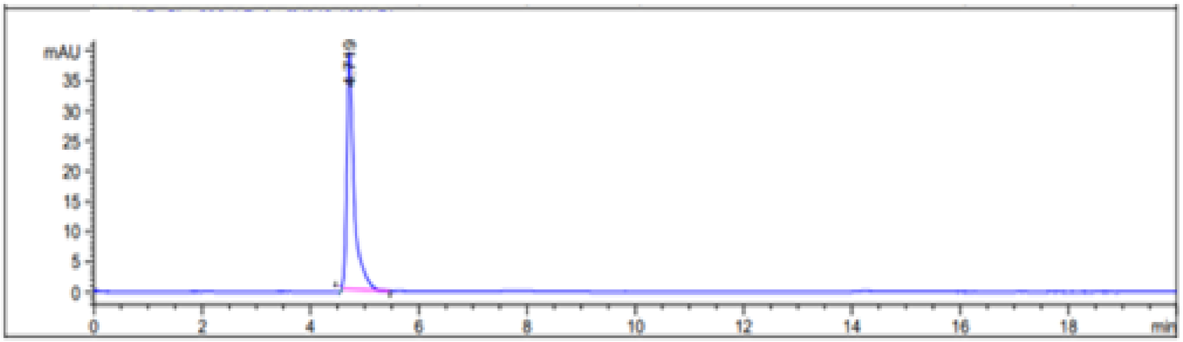

UPLC profile of [G10f]-SHa (**3**) at C-4 reverse-phase column, ACN:H_2O:TFA (60%:40%:0.082%)

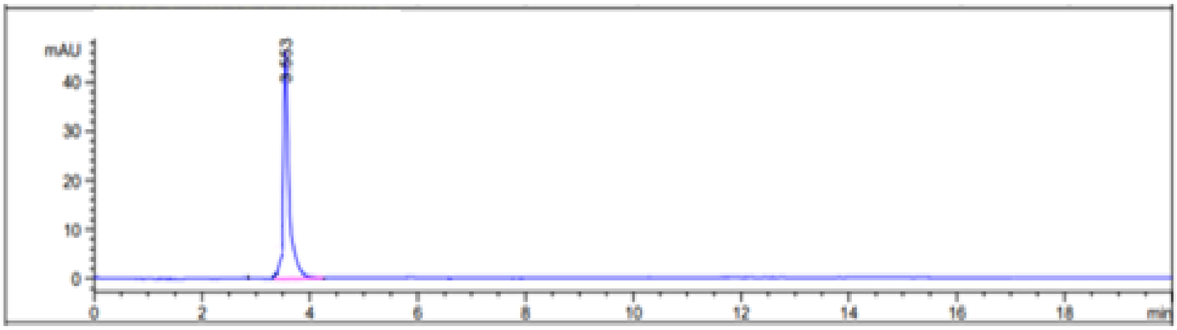

UPLC profile of [G10K]-SHa (**4**) at C-4 reverse-phase column, ACN:H_2O:TFA (60%:40%:0.082%)

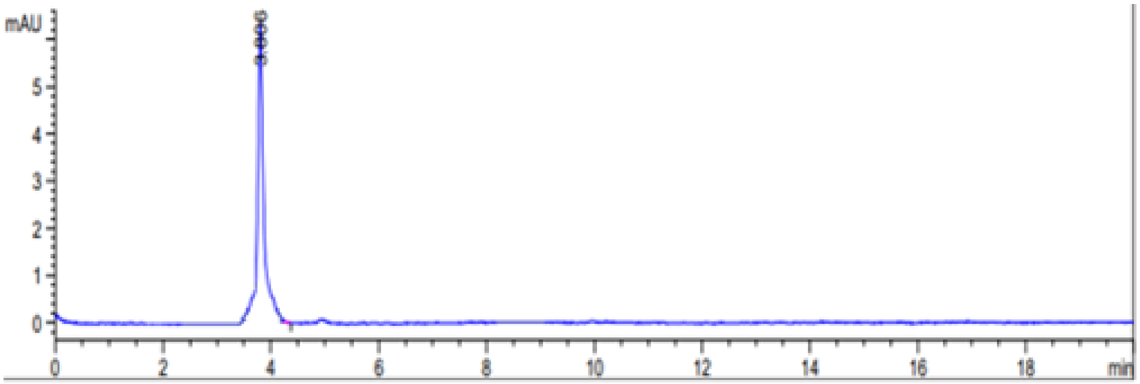

UPLC profile of [G10n]-SHa (**5**) at C-4 reverse-phase column, ACN:H_2O:TFA (60%:40%:0.082%)

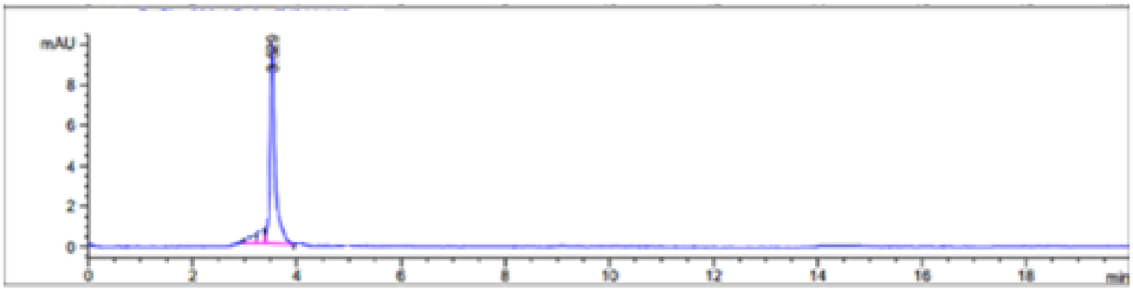

UPLC profile of [G10y]-SHa (**6**) at C-4 reverse-phase column, ACN:H_2O:TFA (60%:40%:0.082%)

Figure-S1: UPLC profile of temporin-SHa peptide (**1**), [G10a]-SHa peptide (**2**) and its second-generation analogs (**3–6**) in ACN : H_2O : TFA (60 : 40 : 0.08) at 215 nm

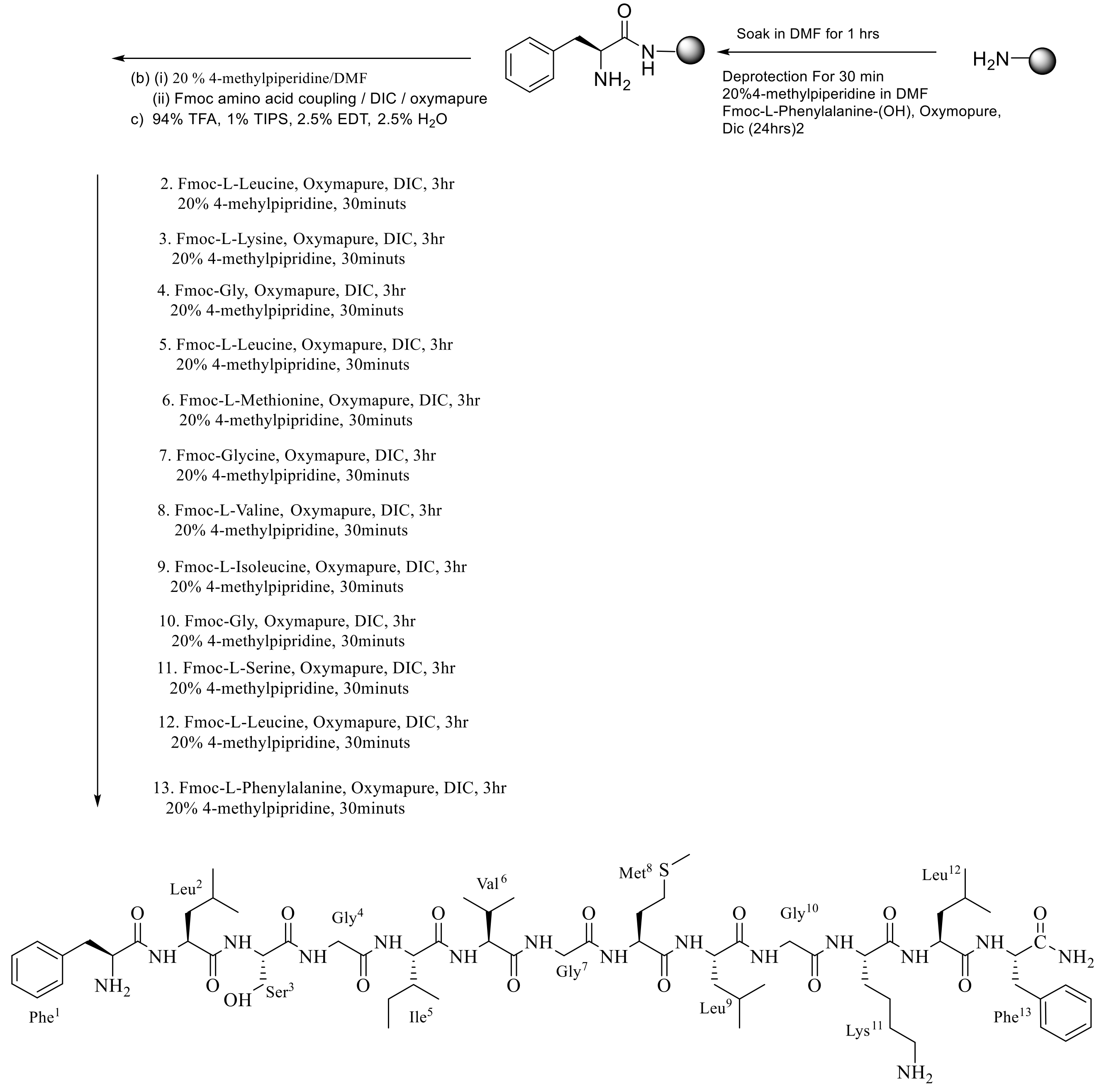

Chemical Formula: $C_{67}H_{109}N_{15}O_{14}S$

Exact Mass: 1379.800

Molecular Weight: 1380.760

Figure-S2: Synthetic scheme of temporin-SHa (**1**)

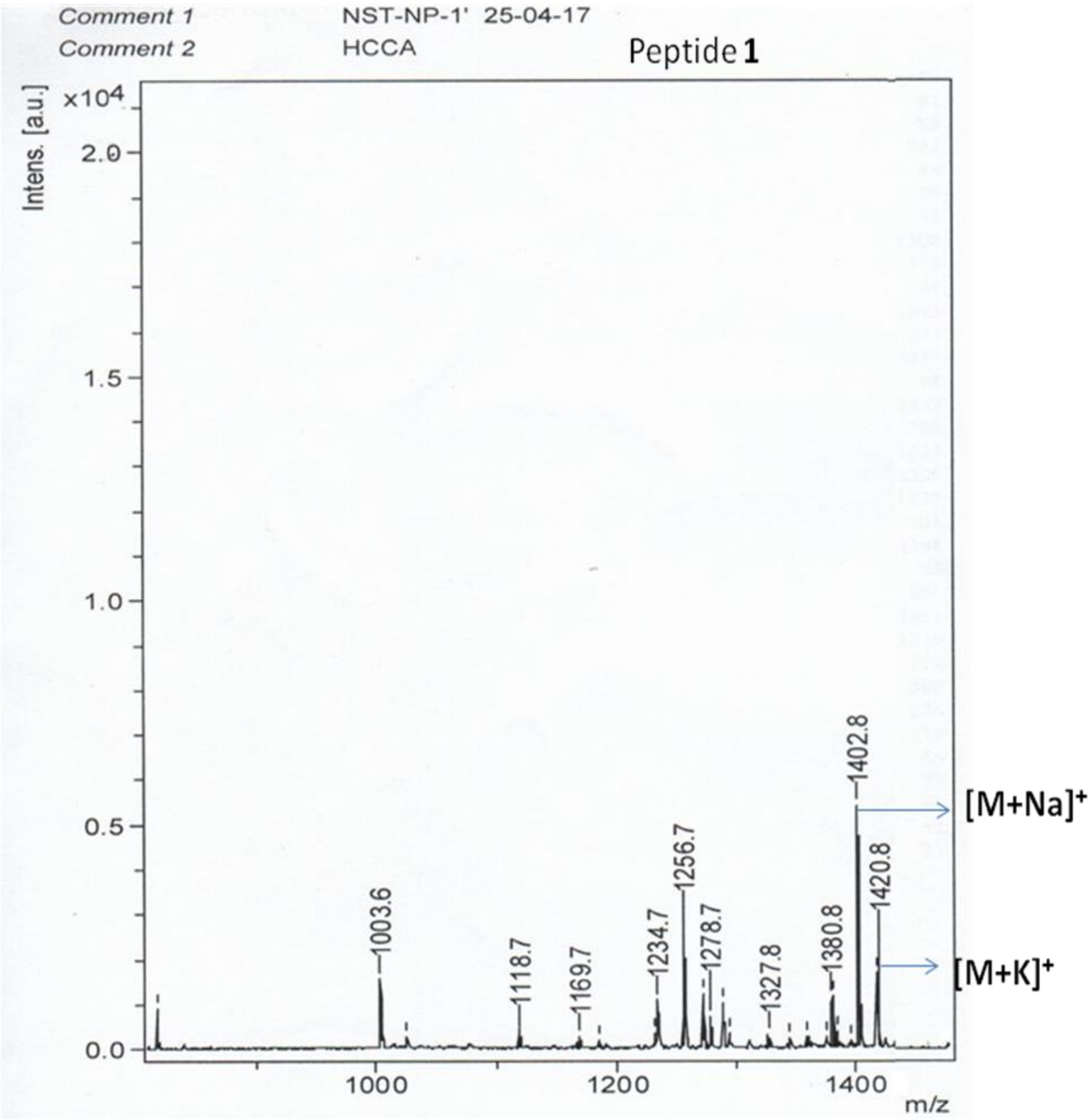

Figure-S3: MALDI mass spectrum of temporin-SHa (**1**)

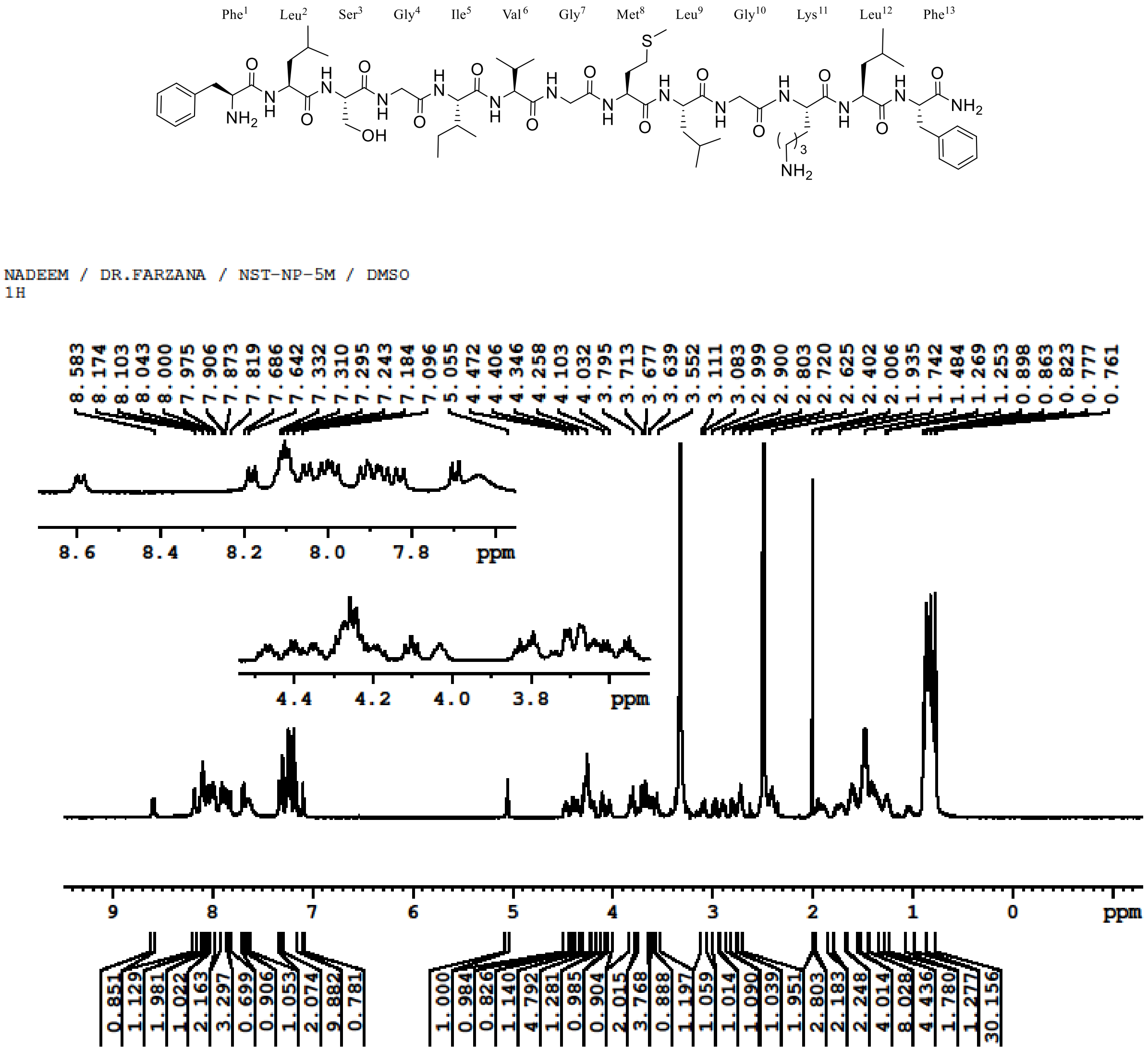

Figure-S4: ^{1}H NMR spectrum of temporin-SHa (**1**)

Residue	Position	Temporin-SHa (1)		
		^{1}H-NMR	*J* (Hz)	^{13}C-NMR
Phe[1]	α	4.04 s		53.4
	β	2.88 dd, 3.09 dd	8.2, 14.2 & 4.38, 14.1	37.1
	1			134.9
	2-6	(7.17-7.18)m		126.2-129.5
	CO			167.7
	NH_2	overlapped		
*Leu[2]	α	4.46 dd	8.5, 14.0	51.0
	β	(1.35-1.49) m		41.0
	γ	(1.62-1.38) m		24.1
	δ_1 CH_3	(0.75-0.80) m		21.5
	δ_2 CH_3	(0.80-0.90) m		23.1
	CO			169.
	NH	8.61 d	8.0	
Ser[3]	CH	4.27 m		55.2
	CH2	3.61p, 3.55p	5.5, 5.6	61.8
	CO			171.18
	NH	8.18 d	7.4	
	OH	5.06t	5.5	
*Gly[4]	α	3.82m, 3.67m		42.0-41.8
	CO			171.2
	NH	8.11 m		
Ile[5]	α	4.24 m		56.7
	β	1.48* m		26.7
	γ	1.69 m		36.5
	δ1	0.79 m		14.7
	δ2	0.76 m		10.9
	CO			168.2
	NH	7.83 d	8.5	
Val[6]	α	4.10 t	7.5	58.0
	β	1.93 m		30.3
	γ	(0.80-0.82) m		18.4, 19.1
	CO			170.6
	NH	7.88 m		
Gly[7]	α	3.82m, 3.67m		42.0-41.8
	CO			
	NH	8.1 m		
Met[8]	α	4.37 m		52.0
	β	2.39 m		29.4
	γ	(1.61-1.47) m		31.6
	δ	1.99 s		15.3
	CO			171.1

	NH	7.98 m		
*Leu[9]	α	4.25 m		51.3
	β	(1.35-1.49) m		40.3
	γ	(1.62-1.38) m		24.1
	δ_1 CH_3	(0.75-0.80) m		21.5
	δ_2 CH_3	(0.80-0.90) m		23.1
	CO			171.1
	NH	8.09 m		
Gly[10]	α	3.82m, 3.67m		42.0-41.8
	CO			168.9
	NH	8.10 m		
Lys[11]	α	4.23 dd	8.5, 13.5	51.9
	β	(1.40-1.47) m		32.0
	γ	1.24 m		22.1
	δ	1.50 m		26.6
	ϕ	2.71 m		39.0
	CO			167.6
	NH	8.06 d	7.6	
	NH_2	overlapped		bs
*Leu[12]	α	4.22 m		51.1
	β	(1.35-1.49) m		40.6
	γ	(1.62-1.38) m		24.1
	δ_1 CH_3	(0.75-0.80) m		21.5
	δ_2 CH_3	(0.80-0.90) m		23.1
	CO			171.07
	NH	7.98 m		
Phe[13]	α	4.42 seq	5.5, 8.5	53.1
	β	2.80 dd, 2.99 dd	8.04, 14.1 & 8.7, 13.7	37.6
	1			137.7
	2-6	(7.22-7.25) m		126.2-129.5
	CO			170.70
	NH	7.70 d	8.1	
	NH_2	7.64	bs	

Table-S5. NMR data of temporin-SHa (**1**) in DMSO-d_6

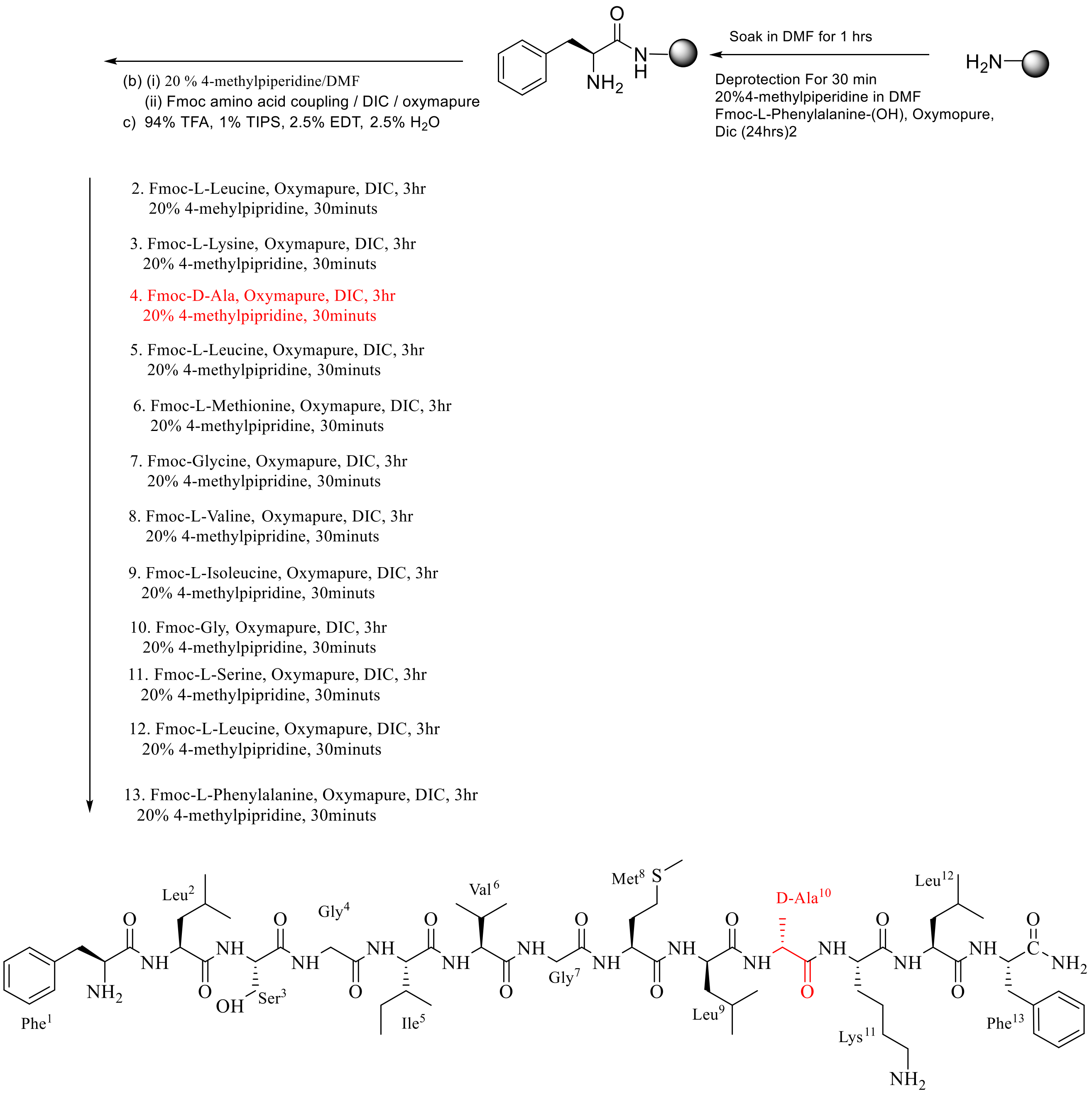

Chemical Formula: $C_{68}H_{111}N_{15}O_{14}S$
Exact Mass: 1393.816
Molecular Weight: 1394.787

Figure-S6. Synthesis of [G10a]-SHa (**2**)

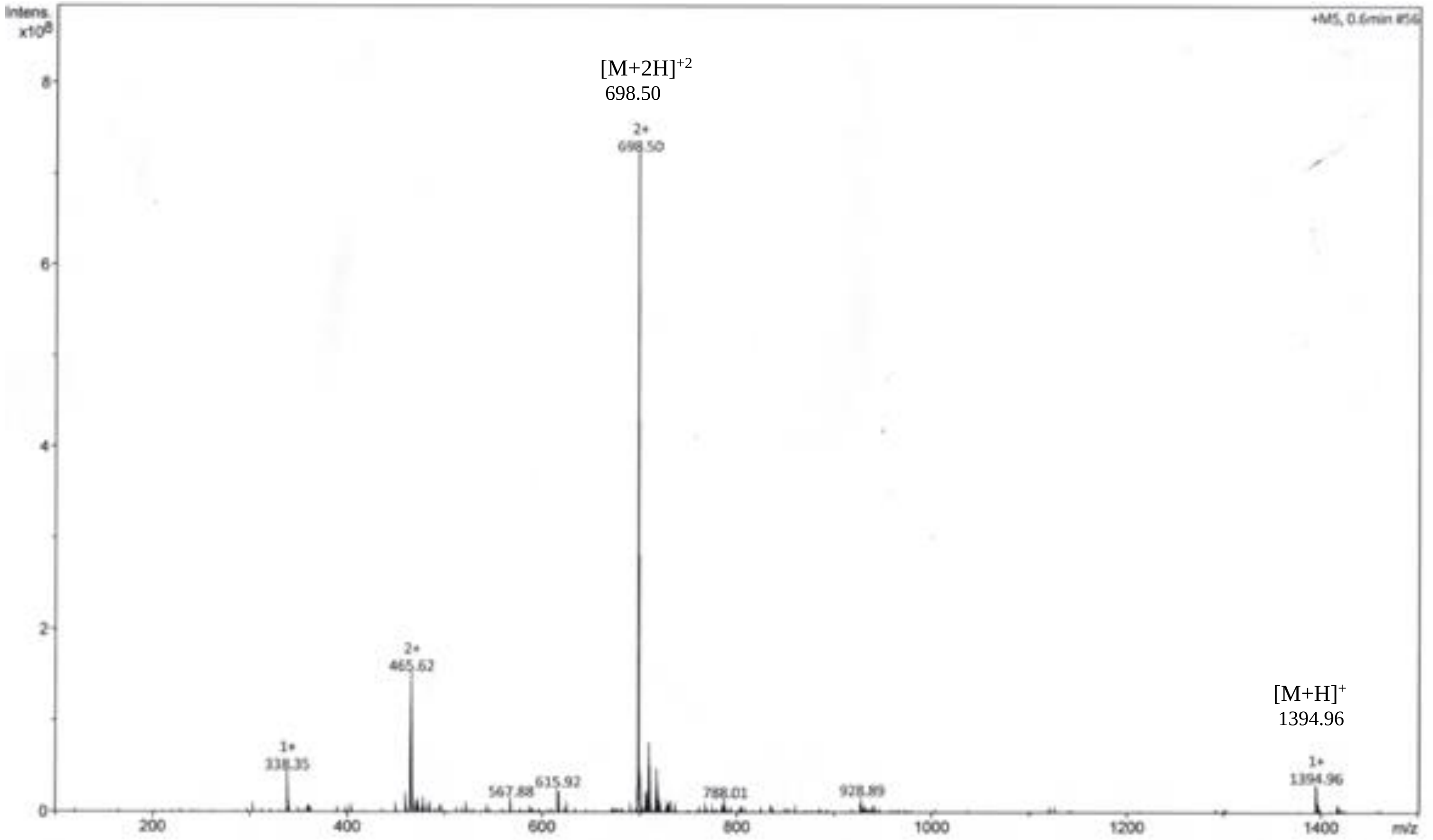

Figure-S7: LR-ESI-MS of [G10a]-SHa (**2**)

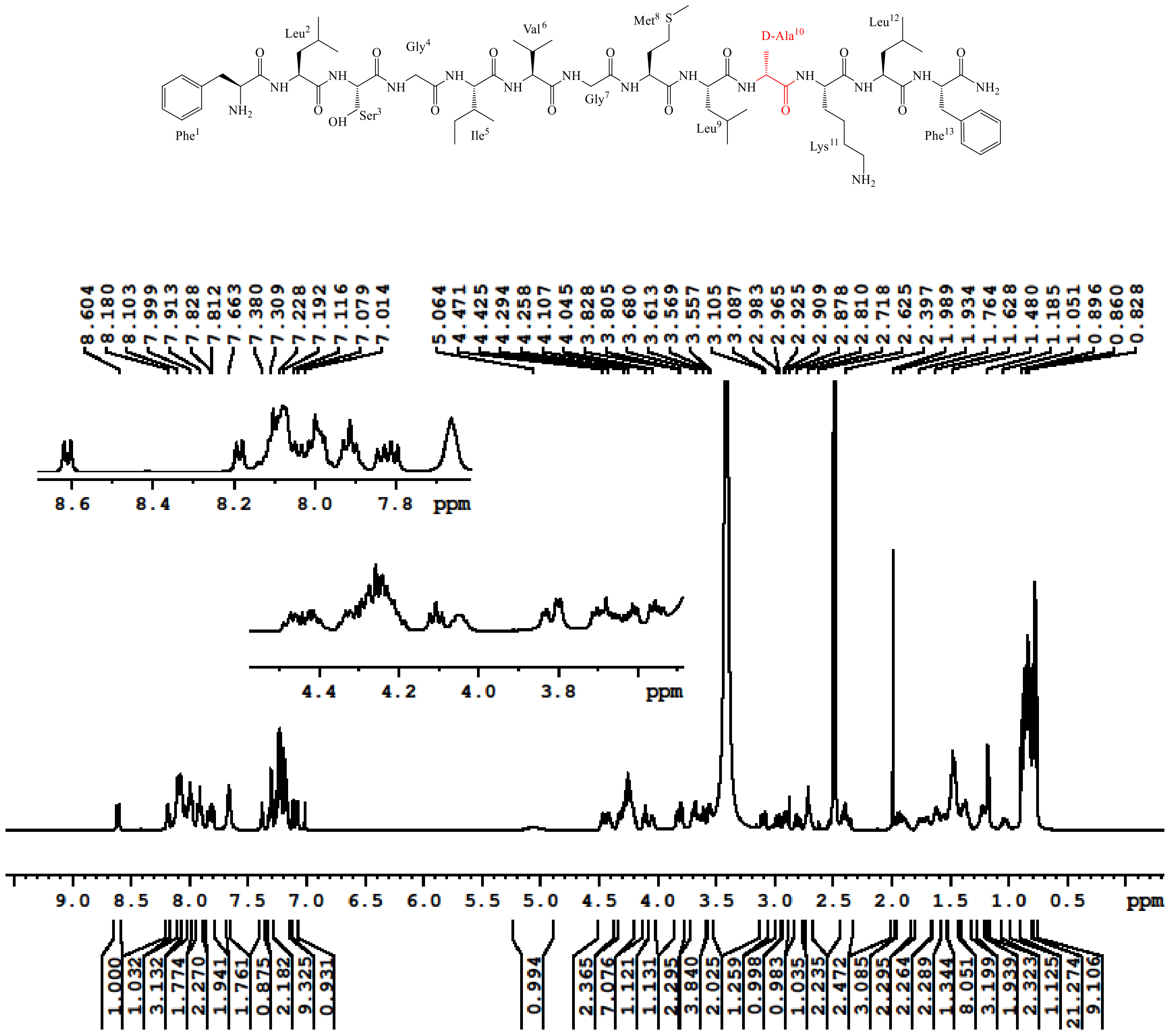

Figure-S8: ^{1}H-NMR of [G10a]-SHa (**2**)

Residue	Position	[G10a] SHa (2)		
		600 MHz ^{1}H-NMR	*J* (Hz)	150 MHz ^{13}C-NMR
Phe[1]	α	4.04		53.20
	β	2.87 dd,, 3.07 dd	8.2, 14.2 & 4.38, 14.1	37.00
	1			134.7
	2-6	(7.17-7.18) m		129.2
	CO			167.7
	NH_2			
*Leu[2]	α	4.46 dd	8.22, 14.3	51.0
	β	(1.43-1.55) m		41.0
	γ	(1.61-1.04) m		24.2
	δ_1 CH_3	(0.75-0.80) m		21.5,
	δ_2 CH_3	(0.80-0.90) m		23.0
	CO			169.
	NH	8.6 d	9.3	
Ser[3]	CH	4.26 m		55.1
	CH2	3.60 p, 3.54 p	5.5, 5.5	61.5
	CO			171.18
	NH	8.00 m		
	OH	5.06 t	5.4	
Gly[4]	α	3.82 m, 3.68 m		42.1
	CO			171.2
	NH	8.11 m		
Ile[5]	α	4.24 m		56.7
	β	1.45* m		26.6
	γ	1.69 m		36.5
	δ1	0.79 m		14.6

	δ2	0.76 m		10.9
	CO			168.2
	NH	7.84 d	8.4	
Val[6]	α	4.10 t	7.5	58.0
	β	1.93 m		30.2
	γ	(0.80-0.82) m		18.9, 19.2
	CO			170.6
	NH	7.90 m		
Gly[7]	α	3.67 m, 3.80 m		41.9
	CO			168.9
	NH	7.90 m		
Met[8]	α	4.32 (m)		52.0
	β	2.39 m		29.4
	γ	(1.75-1.85)m		31.8
	δ	1.97 m		15.3
	CO			171.1
	NH	8.19 d	7.3	
*Leu[9]	α	4.21 m		51.35
	β	(1.43-1.55) m		40.6
	γ	(1.61-1.04) m		24.0
	$δ_1$ CH_3	(0.75-0.80) m		21.6
	$δ_2$ CH_3	(0.80-0.90) m		22.9
	CO			171.1
	NH	8.0 m		
Ala[10]	α	4.25 m		48.31
	CH_3	1.18 d	6.9	18.42
	CO			171.18
	NH	7.92 m		

Lys[11]	α	4.26m		51.1
	β	(1.40-1.47)m		31.30
	γ	1.22 m		22.17
	δ	1.50 m		26.64
	ϕ	2.71 m		38.79
	CO			167.6
	NH	7.99 m		
	NH_2	bs		
*Leu[12]	α	4.22 m		51.4
	β	(1.43-1.55) m		40.4
	γ	(1.61-1.04) m		24.0
	δ_1 CH_3	(0.75-0.80) m		21.5
	δ_2 CH_3	(0.80-0.90) m		23.2
	CO			171.07
	NH	8.07 m		
Phe[13]	α	4.40 dd	8.3, 13.5	53.5
	β	2.78dd , 2.96dd	8.04, 14.1 & 8.7, 13.7	37.6
	1			137.6
	2-6	7.22-7.25m		127.1-128.0
	CO			170.70
	NH	7.81 d	8.4	
	NH_2			

Table S9: ^{1}H NMR Data of [G10a]-SHa (**2**) in d_6-DMSO.

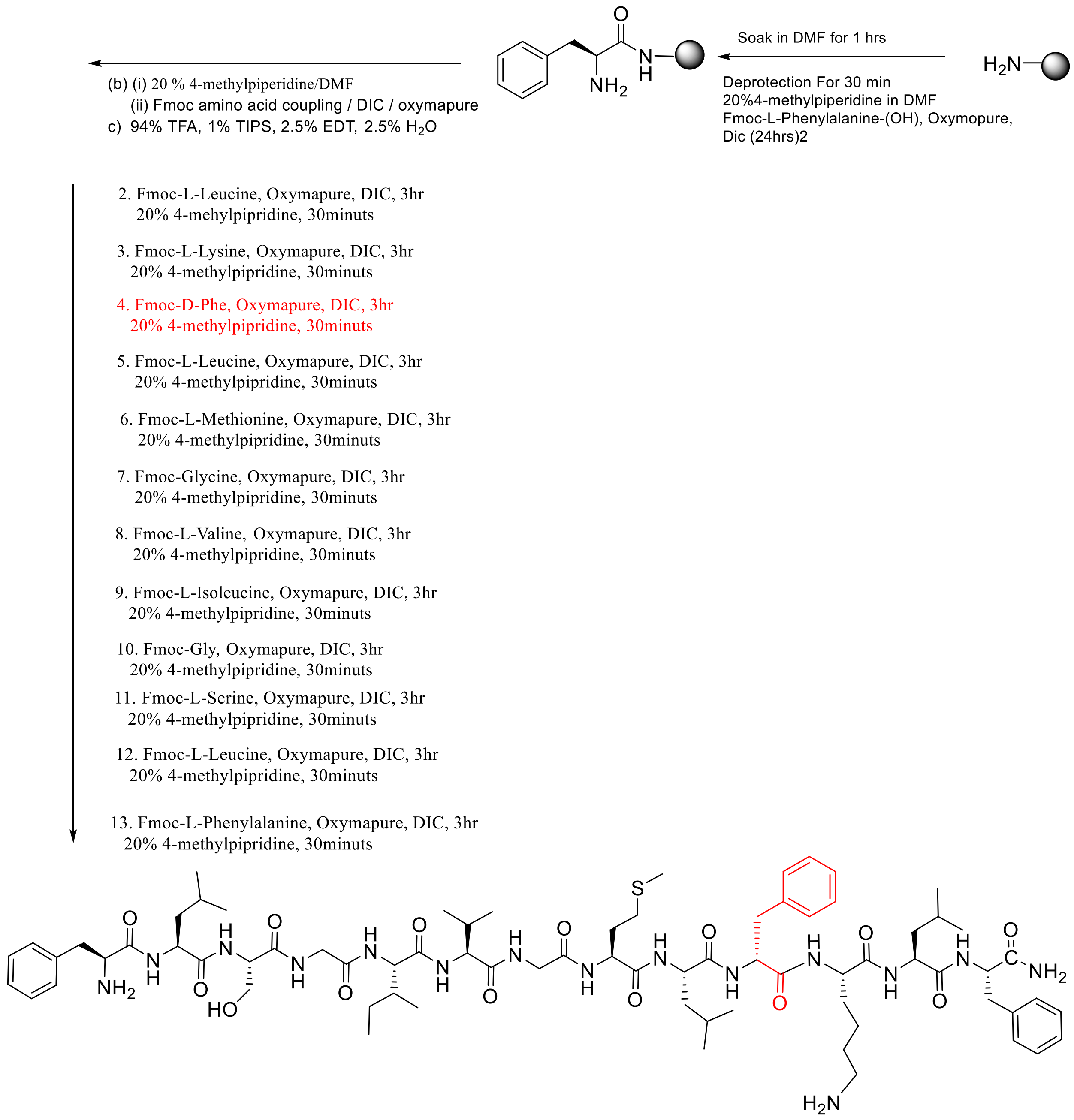

$C_{74}H_{115}N_{15}O_{14}S$
Exact Mass: 1469.8469
Mol. Wt.: 1470.862

Figure-S10: Structure of [G10f]-SHa (**3**)

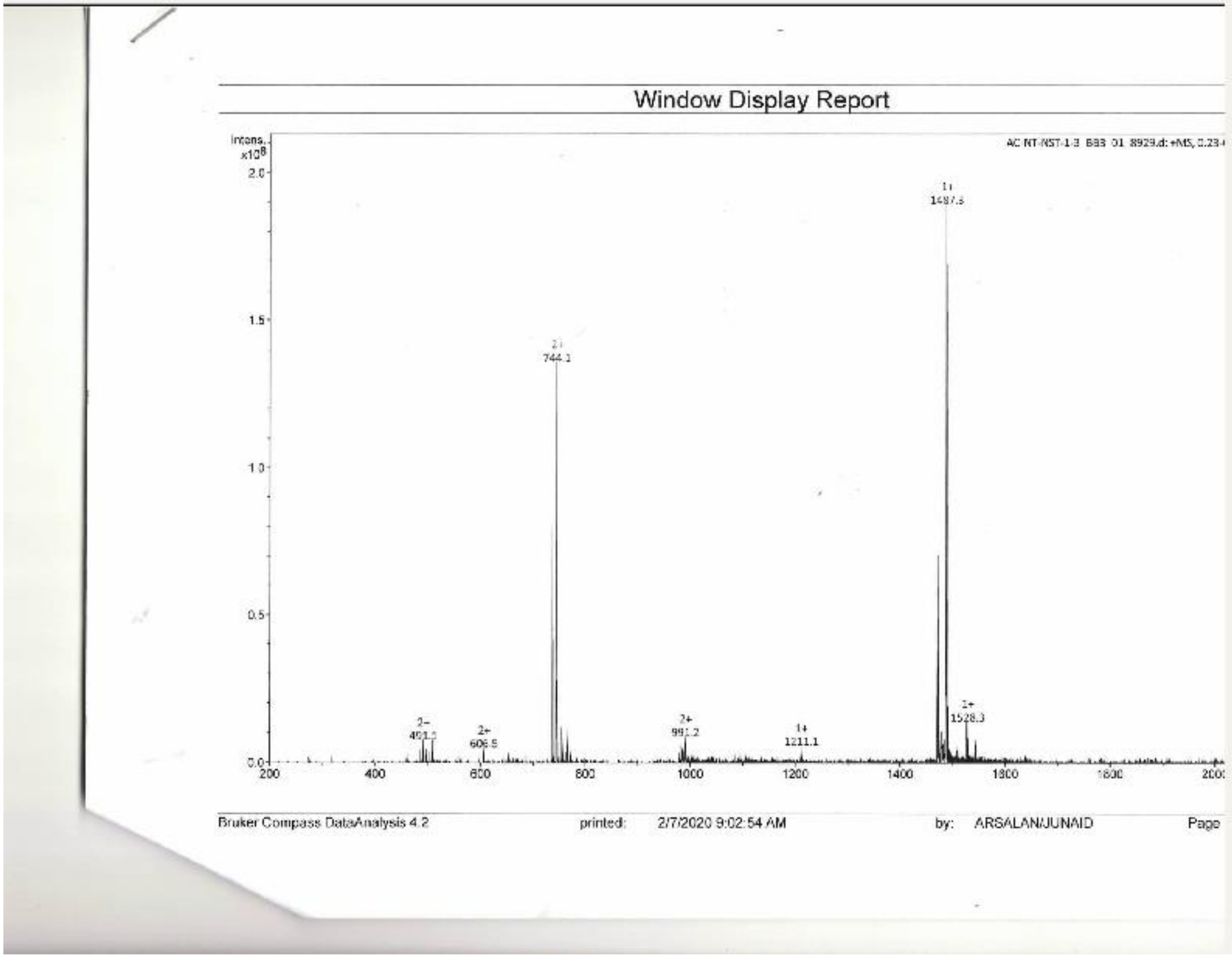

Figure-S11: HR-ESI-MS of [G10f]-SHa (**3**)

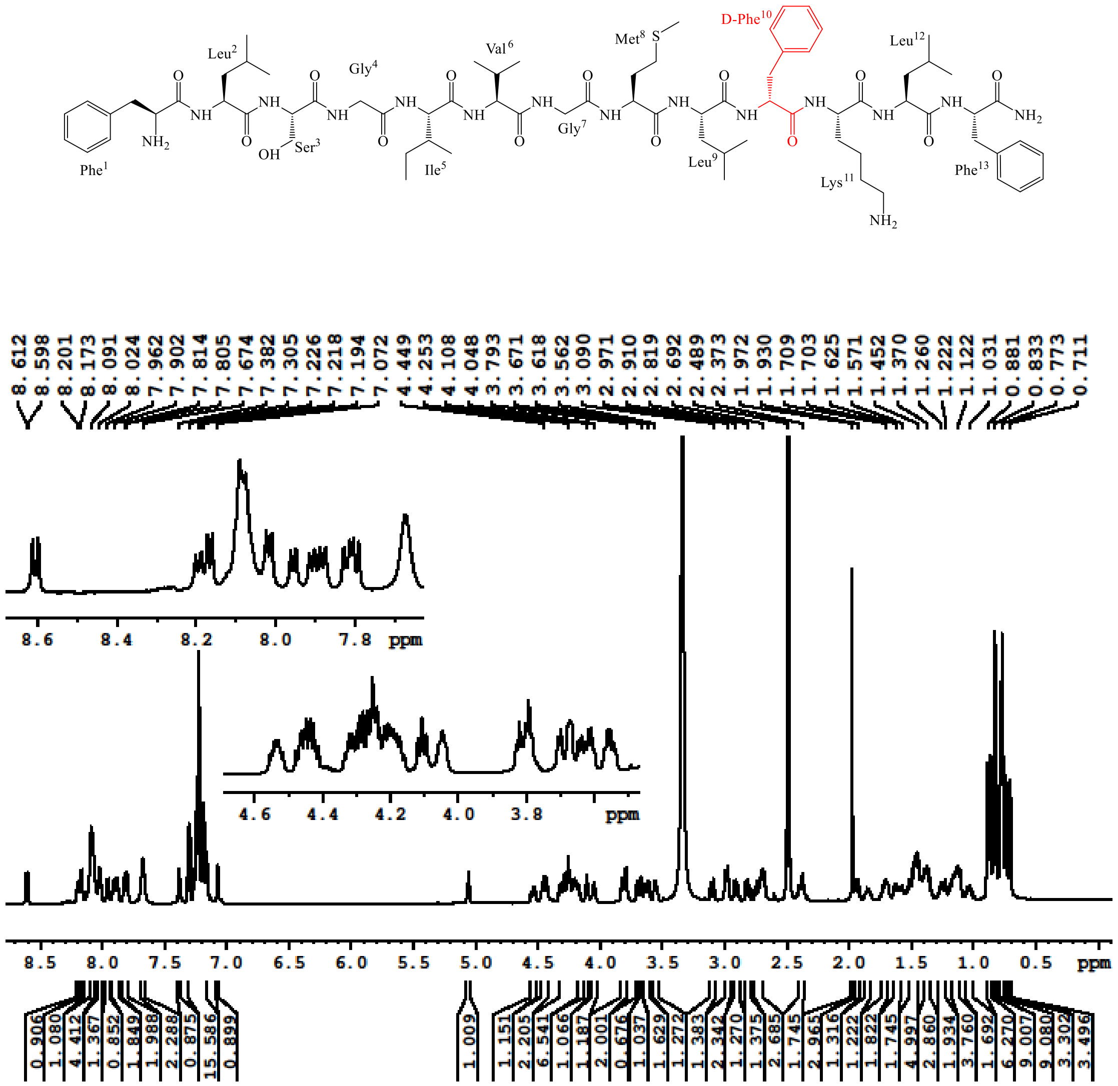

Figure-S12: ^{1}H-NMR of [G10f]-SHa (**3**)

Residue	Position	[G10f]-SHa (3)		
		**^{1}H-NMR (600 MHz)**	*J* (Hz)	**^{13}C-NMR (150 MHz)**
D-Phe[1]	α	4.04 *b signal*		53.0
	β	2.88 *dd*, 3.1 *dd*	8.4, 5.4 and 6.0, 6.1	37.3
	1			134.9
	2-6	(7.15-7.28) *m*		126.2-129.5
	CO			167.7
	NH_2	Signal overlapped		
Leu[2]	α	4.46 *q*	8.9, 4.9 and 9.3	53.8
	β	(1.34-1.49) *m*		40.5
	γ	(1.60-1.61) *m*		23.8-24.5
	CH_3 a	(0.75-0.80) *m*		21.5
	CH_3 b	(0.85-0.89) *m*		23.1
	CO			171.5-171.6
	NH	8.64 *d*	8.2	
*Ser[3]	CH	4.28 *m*		56.5
	CH_2	3.61 *m*, 3.55 *m*		61.1
	CO			170.4
	NH	8.17 *d*	7.3	
	OH	5.05 *t*	5.4	
* Gly[4]	α	3.6-3.8 *m*		41.8
	CO			168.5-170.2
	NH	8.08 *m*		
Ile[5]	α	4.25 *m*		56.5
	β	1.706 *m*		36.3

	γ	1.76 *m*		24.3
	$δ_1$	0.778 *m*		15.4
	$δ_2$	0.77 *m*		11.1
	CO			168.7
	NH	8.08 *d*	9.3	
Val[6]	α	4.10 *m*		57.9
	β	1.94 *m*		30.4
	γ	(0.80-0.82) *m*		18.1
	CO			171.2
	NH	7.88 *d*	8.3	
Gly[7]	α	3.82 *m*, 3.67 *m*		41.8
	CO			168.5-170.2
	NH	8.09 *d*	9.3	
*Met[8]	α	4.31 *m*		51.8
	β	2.37 *m*		29.4
	γ	(1.61-1.47) *m*		31.8
	δ	1.99 *s*		14.9
	CO			170.6
	NH	7.95 *d*	7.5	
*Leu[9]	α	4.21 *m*		51.3
	β	(1.34-1.49) *m*		40.5
	γ	(1.60-1.62) *m*		24.1
	CH_3 a	(0.75-0.80) *m*		21.5
	CH_3 b	(0.80-0.90) *m*		23.1
	CO			171.4
	NH	8.01 *d*	7.7	

*D-Phe[10]	α	4.43		48.2
	β	1.17-1.18		18.1
	1			137.6
	2-6	(7.14-7.28) *m*		126.2-129.5
	CO			172.4
	NH	7.79 *m*		
*Lys[11]	α	4.25 *m*		51.6
	β	(1.40-1.47) *m*		31.6
	γ	1.25 *m*		22.1
	δ	1.47 *m*		29.1
	ϕ	2.71 *m*		38.9
	CO			170.9
	NH	7.82	7.6	
	NH_2	*7.67 b signal*		
*Leu[12]	α	4.18 *m*		51.5
	β	(1.35-1.49) *m*		40.6
	γ	(1.62-1.38) *m*		24.1
	CH_3 a	(0.75-0.80) *m*		21.5
	CH_3 b	(0.80-0.90) *m*		23.1
	CO			167.7
	NH	7.905 *d*		
D-Phe[13]	α	4.54 *dd*		50.8
	β	2.7 *dd*, 2.99 *dd*	8.4, 5.4 and 6.0, 6.1	37.3
	1			137.6
	2-6	(7.14-7.28) *m*		126.2-129.5

	CO			170.7
	NH	8.19 *d*	8.1	
	NH_2	7.07 *s*, 7.38 *s*		

Table-S13: NMR data of [G10f]-SHa (**3**) in *d*$_6$-DMSO

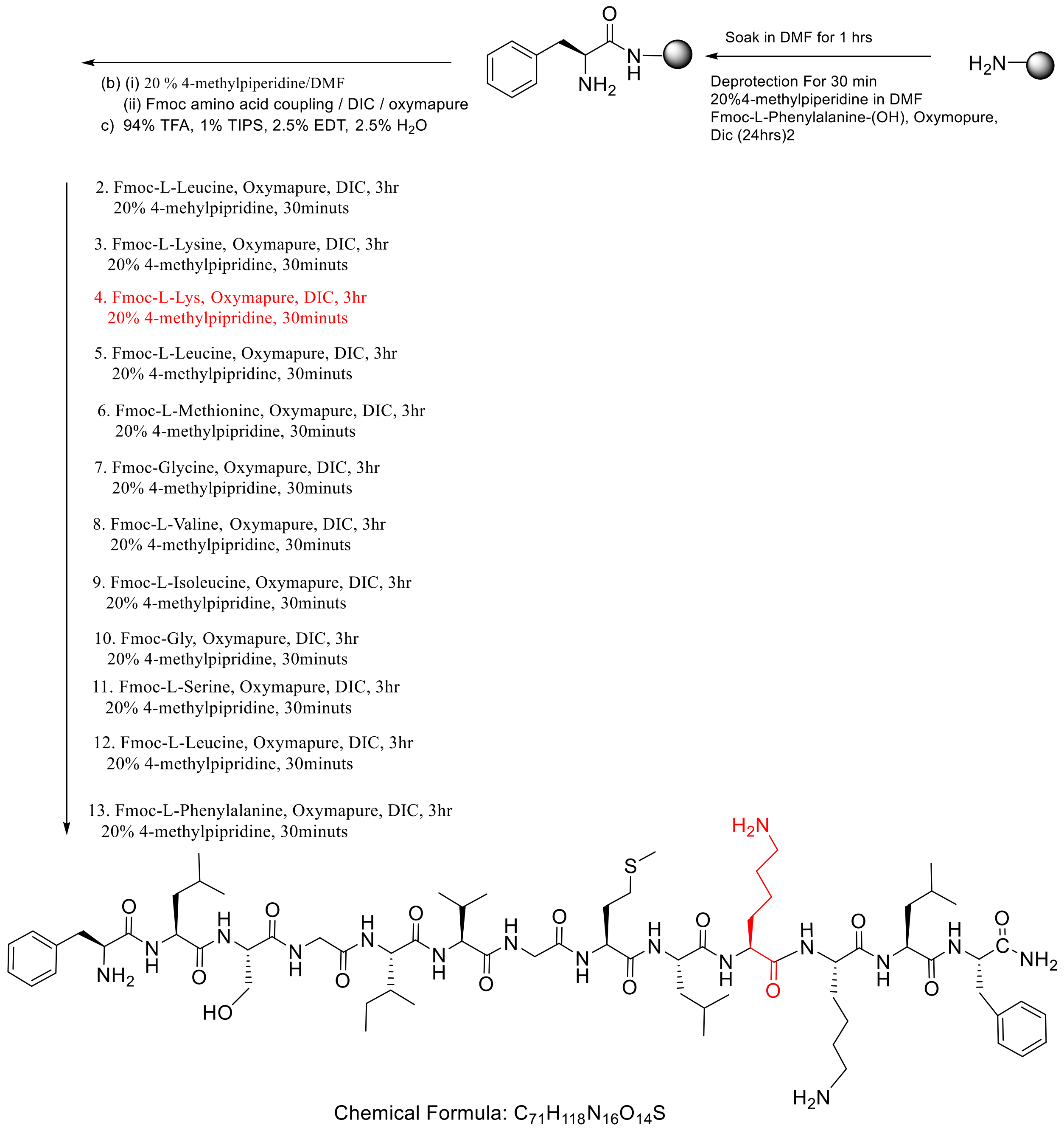

Chemical Formula: $C_{71}H_{118}N_{16}O_{14}S$
Exact Mass: 1450.873
Molecular Weight: 1451.883

Figure-S14: Synthesis of [G10K]-SHa (**4**)

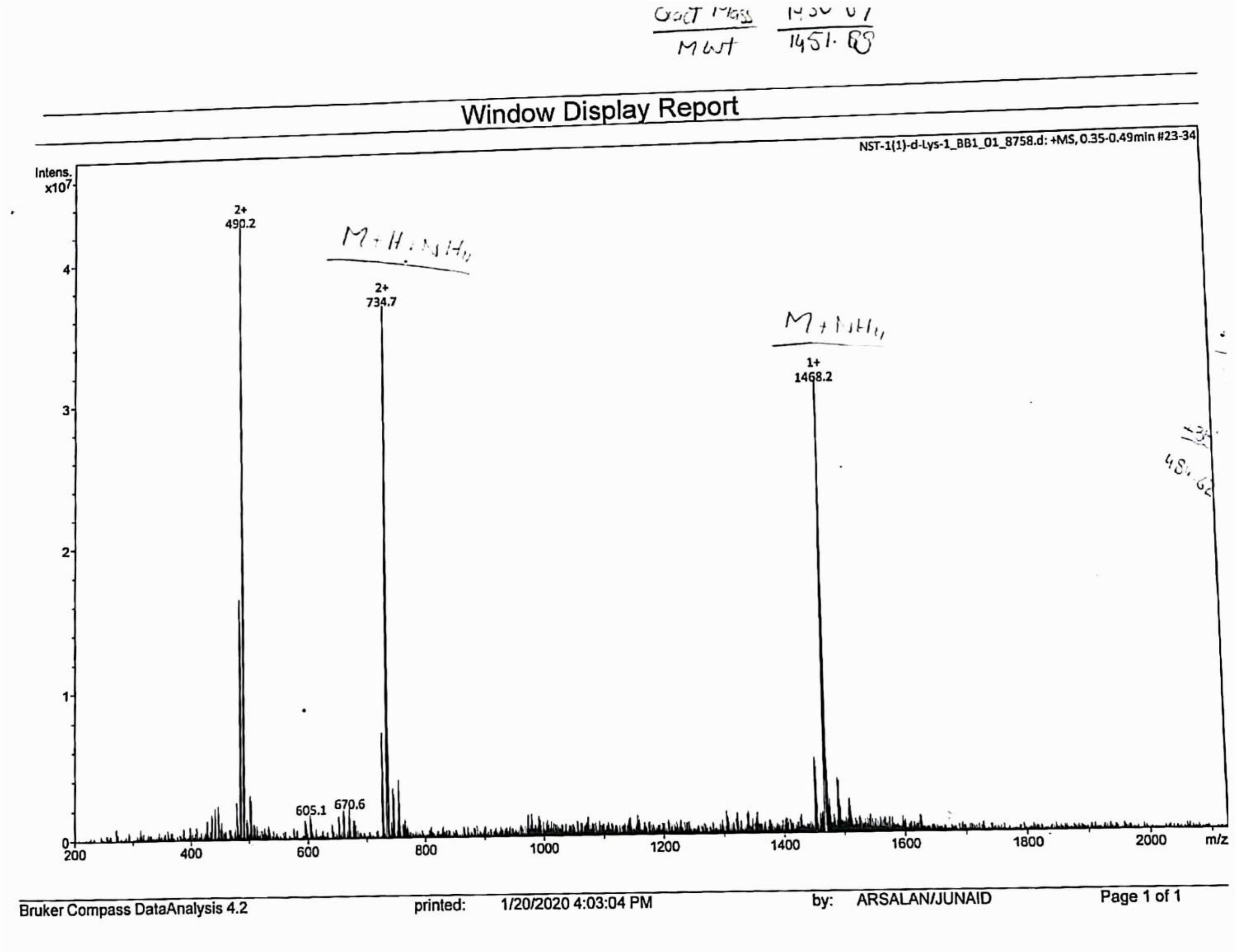

Figure-S15: LR-ESI-MS of [G10K]-SHa (**4**)

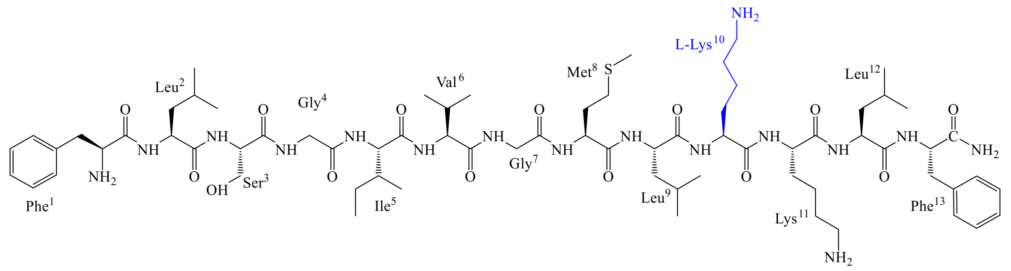

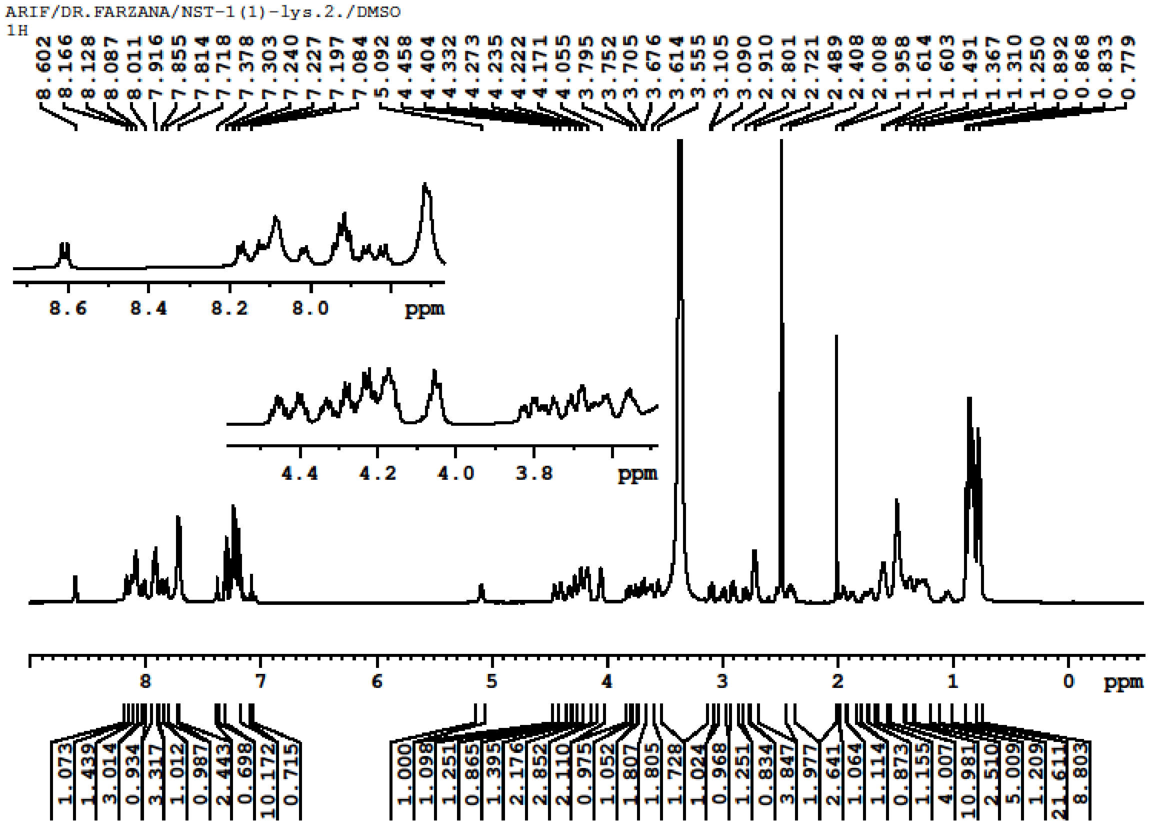

Figure-S16: ^{1}H-NMR spectrum of [G10K]-SHa (**4**)

Residue	Position	[G10K]-SHa (**4**)		
		**^{1}H-NMR (600 MHz)**	*J* (Hz)	**^{13}C-NMR (150 MHz)**
D-Phe[1]	α	4.05 *b signal*		53.1
	β	2.88 *dd*, 3.1 *dd*	4.9, 4.8 and 4.8, 4.6	37.1
	1			134.9
	2-6	(7.15-7.28) *m*		126.2-129.5
	CO			167.8
	NH_2	Signal overlapped		
Leu[2]	α	4.45 *q*	8.6, 5.6 and 8.6	51.1
	β	(1.34-1.49) *m*		41.2
	γ	(1.60-1.61) *m*		24.2
	CH_3 a	(0.75-0.80) *m*		21.5
	CH_3 b	(0.85-0.89) *m*		23.1
	CO			171.5
	NH	8.60 *d*	8.2	
*Ser[3]	CH	4.28 *q*	5.6, 6.7 and 6.0	55.1
	CH2	3.61 *m*, 3.55 *m*		61.1
	CO			169.8-170.6
	NH	8.15 *d*	7.0	
	OH	5.08 *b signal*		
* Gly[4]	α	3.6 *dd*, 3.8 *dd*	5.6, 5.4 and 5.2, 5.0	41.8
	CO			168.5
	NH	8.09 *m*		
Ile[5]	α	4.25 *m*		56.5

	β	1.706 *m*		36.6
	γ	1.76 *m*		24.5
	δ1	0.778 *m*		15.5
	δ2	0.77 *m*		11.1
	CO			171.1
	NH	7.82 *d*	8.2	
Val[6]	α	4.11 *m*	12, 7.8	57.9
	β	1.94 *m*		30.9
	γ	(0.80-0.82) *m*		18.8
	CO			169.8-170.6
	NH	7.88 *d*	8.0	
Gly[7]	α	3.82 *dd*, 3.67 *dd*	5.6, 5.4 and 5.2, 5.0	41.8
	CO			168.8
	NH	8.03 *d*	7.3	
*Met[8]	α	4.32 *q*	9.3, 5.7 and 8.1	51.8
	β	2.37 *m*		29.7
	γ	(1.61-1.47) *m*		31.9
	δ	1.99 *s*		14.9
	CO			170.9
	NH	7.96 *m*		
*Leu[9]	α	4.34 *m*		51.3
	β	(1.34-1.49) *m*		40.5
	γ	(1.60-1.62) *m*		24.1
	CH_3 a	(0.75-0.80) *m*		21.5
	CH_3 b	(0.80-0.90) *m*		23.1
	CO			171.6

	NH	8.10 *d*	5.6	
*L-Lys[10]	α	4.43		53.3
	β	1.17-1.18		37.6
	γ	1.25 *m*		22.3
	δ	1.47 *m*		29.1
	ϕ	2.71 *m*		38.9
	CO			171.2
	NH	7.796		
*Lys[11]	α	4.22 *m*		51.4
	β	(1.40-1.47) *m*		31.6
	γ	1.25 *m*		22.3
	δ	1.47 *m*		29.1
	ϕ	2.71 *m*		38.9
	CO			170.9
	NH	8.01 *m*		
	NH_2	7.71 *b signal*		
*Leu[12]	α	4.17 m		51.0
	β	(1.35-1.49) *m*		40.7
	γ	(1.62-1.38) *m*		24.2
	CH_3 a	(0.75-0.80) *m*		21.5
	CH_3 b	(0.80-0.90) *m*		23.1
	CO			171.2
	NH	7.91 *m*		
D-Phe[13]	α	4.42 *dd*	5.5, 5.7	54.4
	β	2.7 *dd*, 2.99 *dd*	4.9, 4.8 and 4.8, 4.6	37.7

	1			137.5
	2-6	(7.14-7.28) *m*		126.2-128.1
	CO			172.9
	NH	8.11 *m*		
	NH_2	7.08 *s*, 7.38 *s*		

Table-S17: NMR data of [G10K]-SHa (**4**) in d_6-DMSO

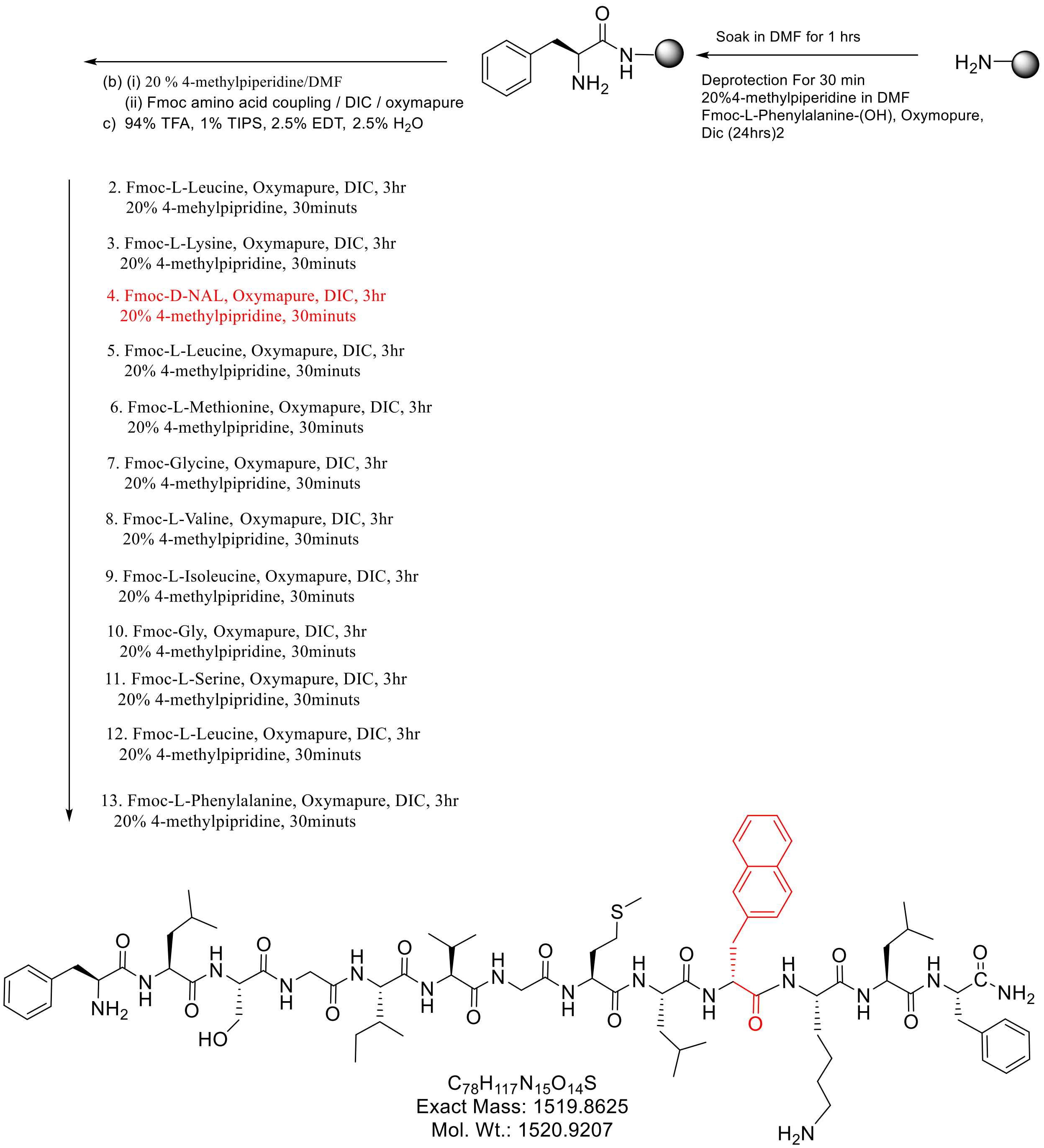

Figure-S18. Synthetic scheme of [G10n]-SHa (**5**)

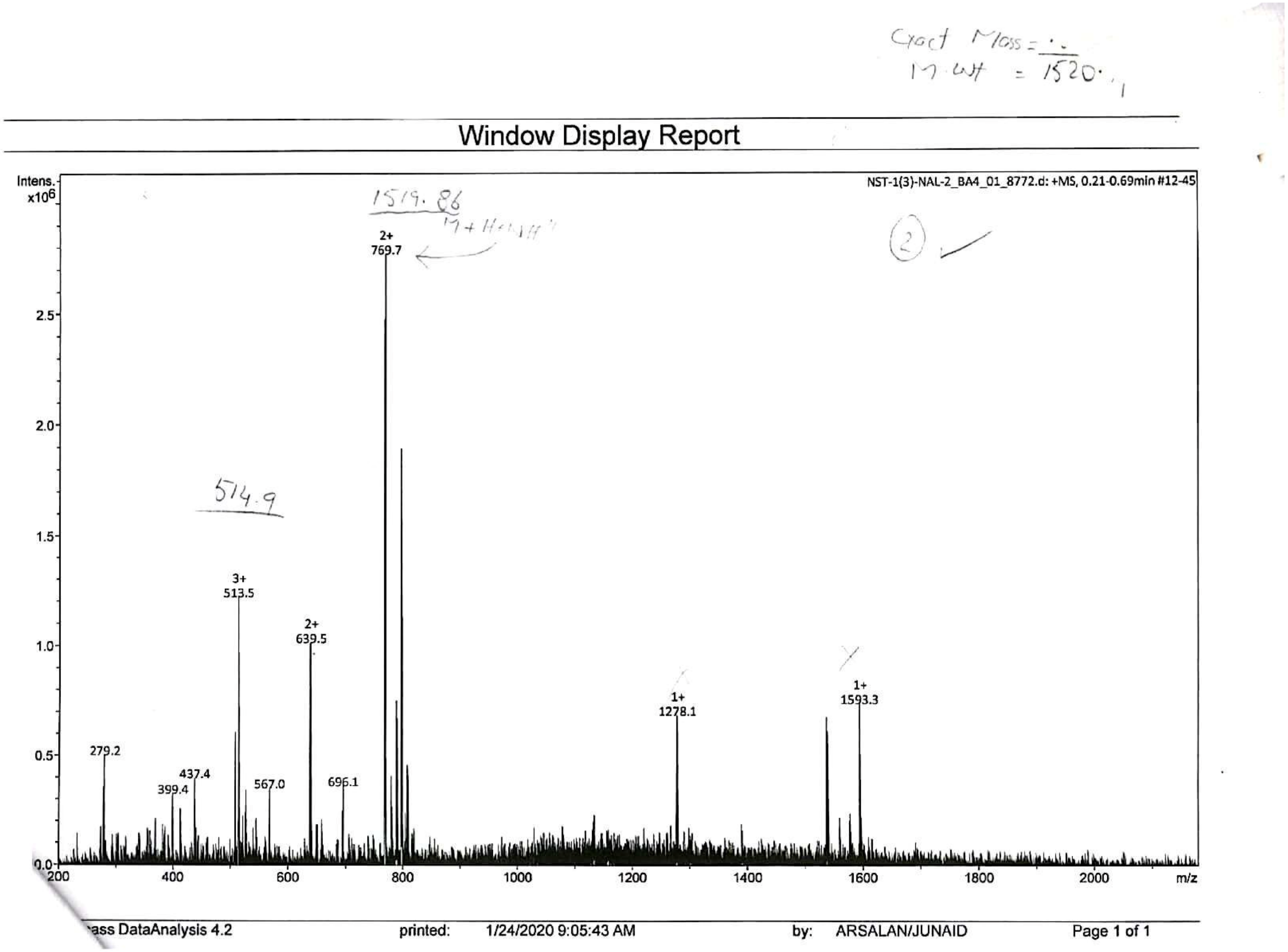

Figure-S19: LR-ESI-MS of [G10n]-SHa (**5**)

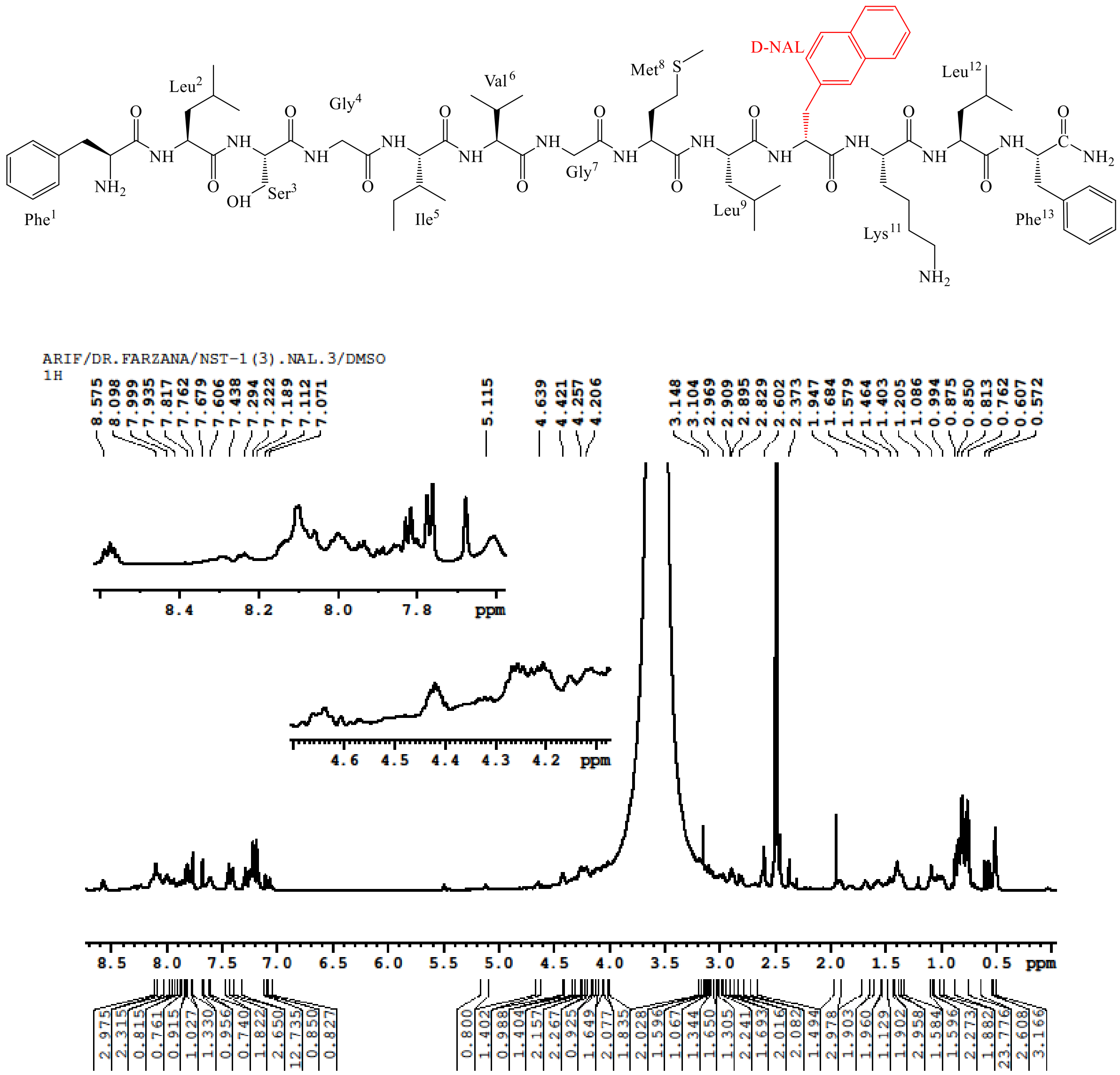

Figure-S20: ^{1}H-NMR of [G10n]-SHa (**5**)

Residue	Position	[G10NAL] SHa (5)		
		**^{1}H-NMR (600 MHz)**	*J* (Hz)	**^{13}C-NMR (150 MHz)**
D-Phe[1]	α	4.01 *b signal*		53.6
	β	2.88 *dd*, 3.1 *dd*	3.6, 4.6 and 4.8, 4.1	37.1
	1			134.9
	2-6	(7.15-7.28) *m*		126.2-129.5
	CO			168.6
	NH_2	Signal overlapped		
Leu[2]	α	4.42 *m*		51.3
	β	(1.34-1.49) *m*		41.2
	γ	(1.60-1.61) *m*		24.2
	CH_3 a	(0.75-0.80) *m*		21.5
	CH_3 b	(0.85-0.89) *m*		23.1
	CO			171.7
	NH	8.58 *m*		
*Ser[3]	CH	4.26 *dd*	3.8, 4.6	55.1
	CH2	3.61 *m*, 3.55 *m*		61.8
	CO			171.8
	NH	8.14 *m*		
	OH	5.08 *t*	5.5	
* Gly[4]	α	3.6-3.8 *m*		41.8
	CO			169.2
	NH	8.09 *m*		
Ile[5]	α	4.21 *m*		56.5

	β	1.706 *m*		36.6
	γ	1.76 *m*		24.5
	δ_1	0.778 *m*		15.5
	δ_2	0.77 *m*		11.1
	CO			171.6
	NH	7.89 *d*	8.1	
Val[6]	α	4.05 *m*		57.9
	β	1.94 *m*		30.9
	γ	(0.80-0.82) *m*		18.8
	CO			171.9
	NH	7.86		
Gly[7]	α	3.82 *m*, 3.67 *m*		41.8
	CO			168.4
	NH	8.10 *m*		
*Met[8]	α	4.32 *m*		51.8
	β	2.37 *m*		29.7
	γ	(1.61-1.47) *m*		31.9
	δ	1.99 *s*		14.9
	CO			170.7
	NH	7.98 *m*		
*Leu[9]	α	4.11 *m*		51.3
	β	(1.34-1.49) *m*		40.5
	γ	(1.60-1.62) *m*		24.1
	CH_3 a	(0.75-0.80) *m*		21.5
	CH_3 b	(0.80-0.90) *m*		23.1
	CO			172.1

	NH	7.84 *m*		
*D-NAL[10]	α	4.64 *m*		53.3
	β	1.17-1.18		37.6
	1			130.6
	2-10	(7.15-7.28) *m*		128.1-155.6
	CO			171.6
	NH	8.23 *m*		
*Lys[11]	α	4.22 *m*		51.4
	β	(1.40-1.47) *m*		31.6
	γ	1.25 *m*		22.3
	δ	1.47 *m*		29.1
	ϕ	2.71 *m*		38.9
	CO			171.6
	NH	8.001 *m*		
	NH_2	7.6 *b signal*		
*Leu[12]	α	4.18 *m*		51.0
	β	(1.35-1.49) *m*		40.7
	γ	(1.62-1.38) *m*		24.2
	CH_3 a	(0.75-0.80) *m*		21.5
	CH_3 b	(0.80-0.90) *m*		23.1
	CO			171.8
	NH	7.99 *m*		
D-Phe[13]	α	4.42 *m*		54.4
	β	2.7 *m*, 2.99 *m*		37.7
	1			137.5

	2-6	(7.14-7.28) *m*		126.2-128.1
	CO			173.0
	NH	7.81 *d*	8.0	
	NH_2	7.07 *b signal*		

Table-S21: NMR Data of [G10n]-SHa (**5**) in d_6-DMSO

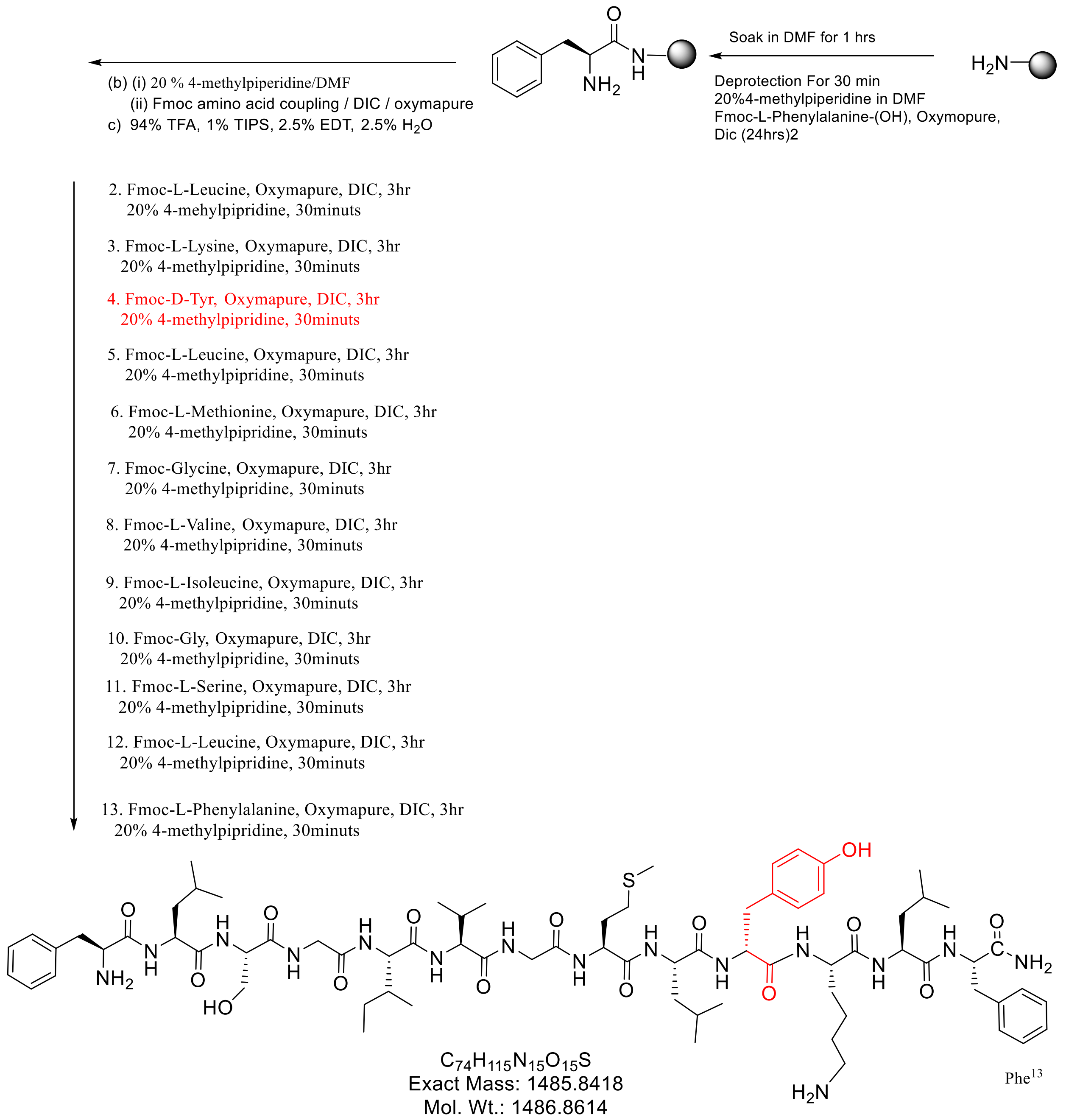

Figure-S22. Synthesis of [G10y]-SHa (**6**)

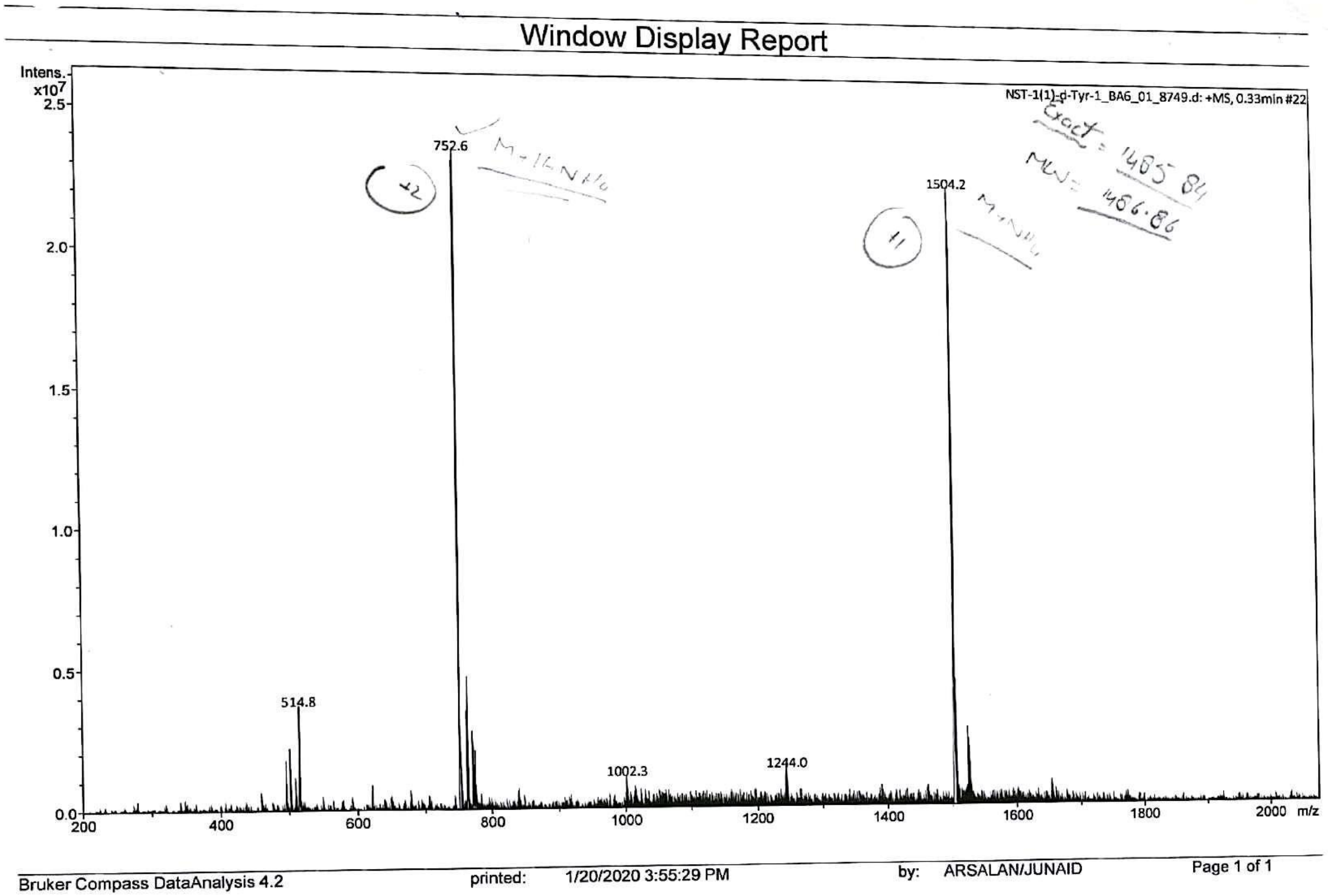

Figure-S23: LR-ESI-MS of [G10y]-SHa (**6**)

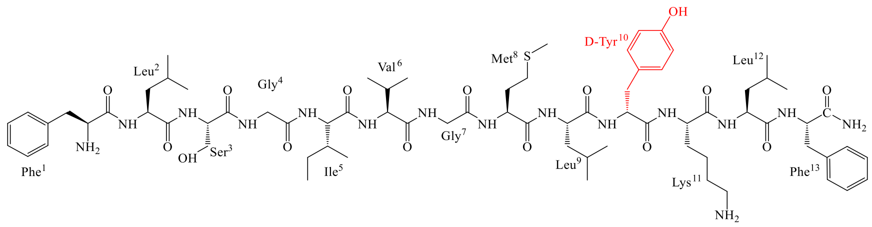

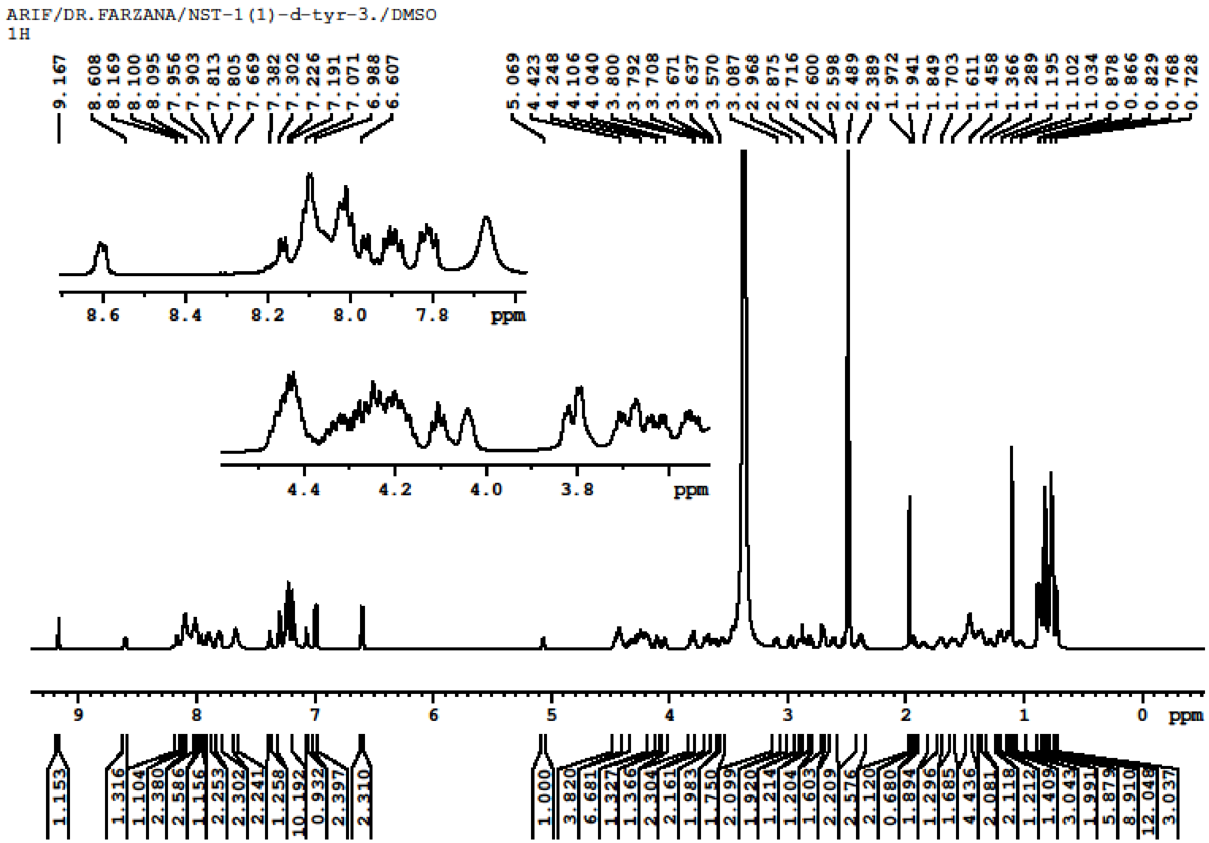

Figure-S24: ^{1}H-NMR of [G10y]-SHa (**6**)

Residue	Position	[G10y]-SHa (6)		
		**^{1}H-NMR (600 MHz)**	*J* (Hz)	**^{13}C-NMR (150 MHz)**
Phe[1]	α	4.05 *b signal*		53.1
	β	2.88 *m*, 3.1 *m*		37.1
	1			134.9
	2-6	(7.15-7.28) *m*		126.2-129.5
	CO			167.8
	NH_2	Signal overlapped		
Leu[2]	α	4.45 *m*		51.1
	β	(1.34-1.49) *m*		41.2
	γ	(1.60-1.61) *m*		24.2
	CH_3 a	(0.75-0.80) *m*		21.5
	CH_3 b	(0.85-0.89) *m*		23.1
	CO			171.5
	NH	8.60 *m*		
*Ser[3]	CH	4.28 *m*		55.1
	CH_2	3.61 *m*, 3.55 *m*		61.1
	CO			169.8-170.6
	NH	8.15 *d*	7.2	
	OH	5.08 *t*	5.2	
* Gly[4]	α	3.6-3.8 *dd*	5.04, 4.5 and 5.7, 4.8	41.8
	CO			168.5
	NH	8.09 *m*		
Ile[5]	α	4.25 *m*		56.5
	β	1.706 *m*		36.6
	γ	1.76 *m*		24.5
	δ_1	0.778 *m*		15.5
	δ_2	0.77 *m*		11.1
	CO			171.1
	NH	7.82 *m*		
Val[6]	α	4.11 *m*		57.9
	β	1.94 *m*		30.9
	γ	(0.80-0.82) *m*		18.8
	CO			169.8-170.6
	NH	7.88 *m*		
Gly[7]	α	3.82 *dd*, 3.67 *dd*	5.04, 4.5 and 5.7, 4.8	41.8
	CO			168.8

	NH	8.03 *m*		
*Met[8]	α	4.31 *m*		51.8
	β	2.37 *m*		29.7
	γ	(1.61-1.47) *m*		31.9
	δ	1.99 *s*		14.9
	CO			170.9
	NH	7.96 *d*	7.5	
*Leu[9]	α	4.34 *m*		51.3
	β	(1.34-1.49) *m*		40.5
	γ	(1.60-1.62) *m*		24.1
	CH_3 a	(0.75-0.80) *m*		21.5
	CH_3 b	(0.80-0.90) *m*		23.1
	CO			171.6
	NH	8.10 *m*		
*D-Tyr[10]	α	4.43		53.3
	β	2.9 *dd* -3.1 *m*		37.6
	1			115.2
	2-6	7.01 *d*	8.3	114.7-130.1
	3-5	6.60 *d*	8.2	114.7-130.1
	4			155.7
	CO			171.2
	NH	7.79 *m*		
	OH	9.17 *b signal*		
*Lys[11]	α	4.22 *m*		51.4
	β	(1.40-1.47) *m*		31.6
	γ	1.25 *m*		22.3
	δ	1.47 *m*		29.1
	ϕ	2.71 *m*		38.9
	CO			170.9
	NH	8.01 *m*		
	NH_2	7.67 *b signal*		
*Leu[12]	α	4.17 *m*		51.0
	β	(1.35-1.49) *m*		40.7
	γ	(1.62-1.38) *m*		24.2
	CH_3 a	(0.75-0.80) *m*		21.5
	CH_3 b	(0.80-0.90) *m*		23.1
	CO			171.2
	NH	7.91 *d*		
Phe[13]	α	4.42 *dd*		54.49

	β	2.7 *m*, 2.99 *m*		37.7
	1			137.5
	2-6	(7.14-7.28) *m*		126.2-128.1
	CO			172.9
	NH	8.11 *m*		
	NH_2	7.07 *s*, 7.38 *s*		

Table-S25: NMR data of [G10y] SHa (**6**) in d_6-DMSO

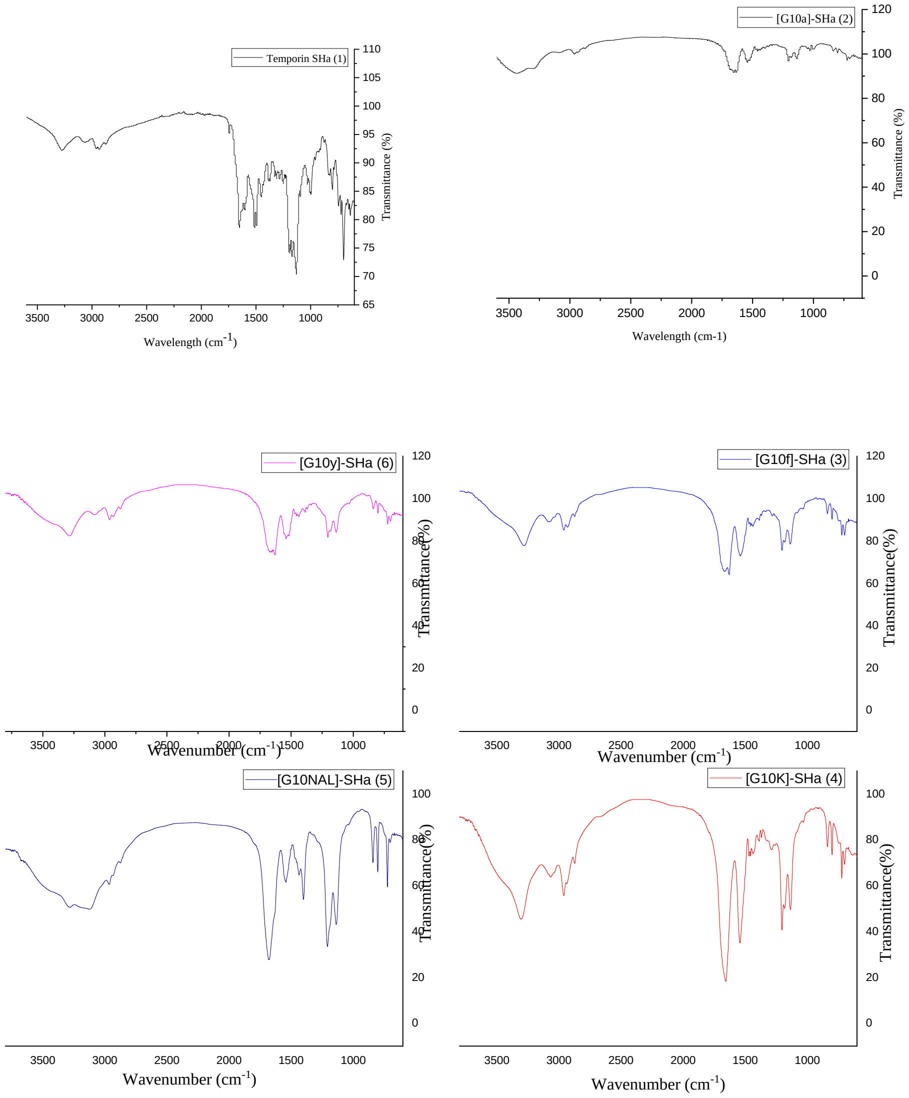

Figure S26: FT-IR spectra of **1-6**

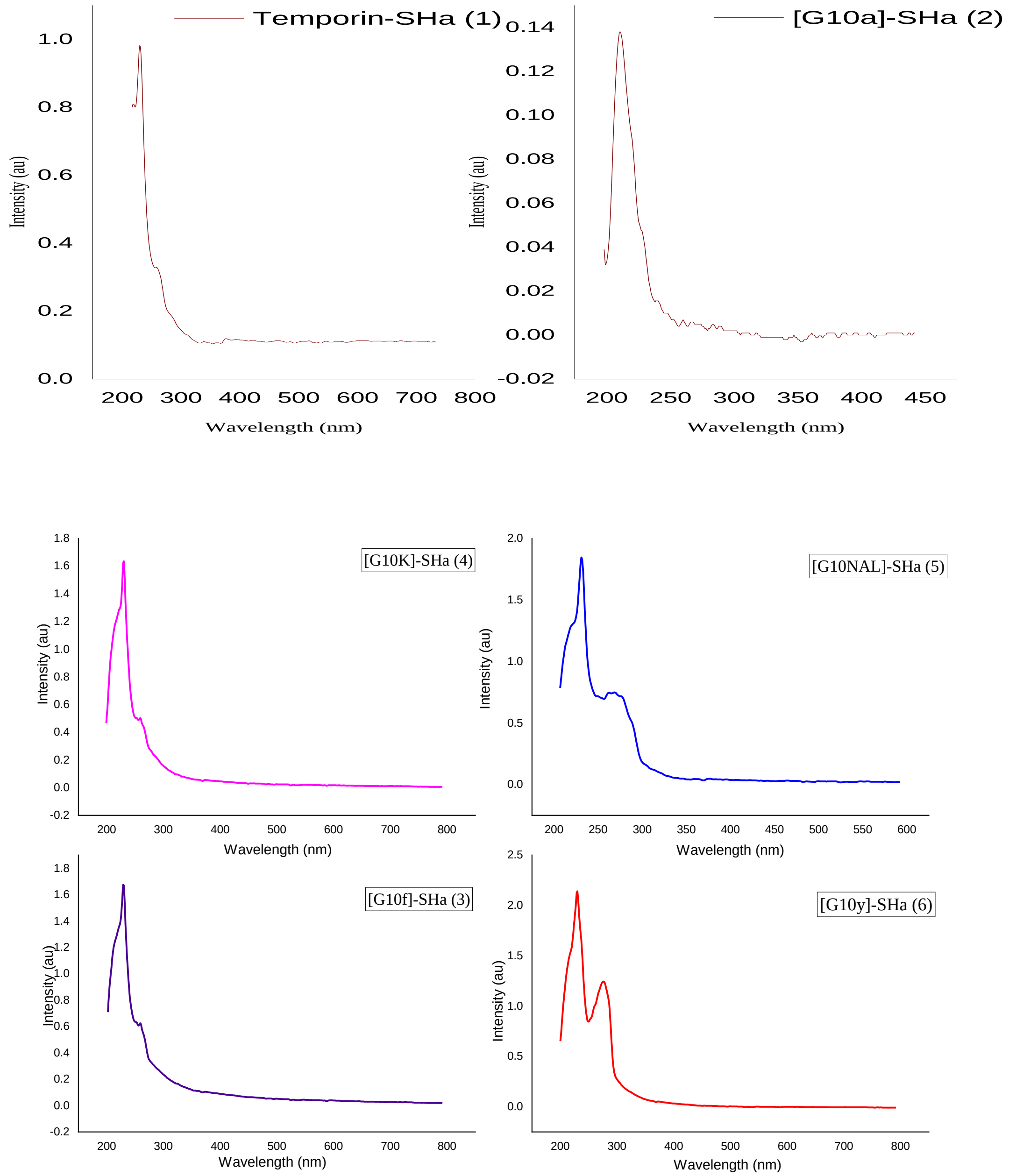

Figure S27: UV-Visible spectra of **1-6** showing peptide bond and disulfide bond region
